# Automated and Artificial Intelligence (AI)-Derived Performance Assessment in Surgical Simulation: A Systematic Review

**DOI:** 10.7759/cureus.100477

**Published:** 2025-12-31

**Authors:** Ahmad Khalifa, Owais Tahhan, Mohammed Albazooni, Mohammed Saeed, Ruha Hamdi, Megan Stanners, Amman Malik, Adnan Malik

**Affiliations:** 1 Surgery, University Hospitals Birmingham NHS Foundation Trust, Birmingham, GBR; 2 Ophthalmology, Midland Metropolitan University Hospital, Smethwick, GBR; 3 Cardiology, Tameside and Glossop Integrated Care NHS Foundation Trust, Ashton-under-Lyne, GBR; 4 Radiology, Dudley Group NHS Foundation Trust, Dudley, GBR; 5 Dermatology, Faculty of Medicine, Aleppo University, Aleppo, SYR; 6 Psychiatry, Midland Metropolitan University Hospital, Smethwick, GBR; 7 Breast Surgery, Glenfield Hospital, Leicester, GBR; 8 Surgery, Sandwell and West Birmingham Hospitals NHS Trust, Birmingham, GBR

**Keywords:** artificial intelligence, automated assessment, competency-based education, computer vision, force sensors, motion tracking, skill evaluation, surgical simulation, surgical training, virtual reality

## Abstract

Artificial intelligence (AI)-assisted and automated performance assessment is increasingly being incorporated into surgical education, yet the degree, efficacy, and trustworthiness of this performance assessment are unknown. A systematic review of the literature published between 2010 and 2025 on PubMed, Scopus, Embase, and IEEE Xplore (including conference proceedings and gray literature) was completed to identify experimental and observational studies that reported the use of algorithms or automated methods of assessment in technical skill assessment in both simulator-based training and real clinical practice. Information was extracted on study characteristics, algorithm type, task complexity, performance measures, evidence of validity and reliability, and the quality of studies, assessed using relevant tools, with results reported descriptively and trends reviewed by study type, year, country, and task domain. Twenty-nine studies met the inclusion criteria, with most using supervised learning algorithms to evaluate technical skills; performance measures ranged widely, and studies were inconsistent in documenting the validity and reliability of the assessments. Few studies presented a real-time adaptive feedback system. The literature reflects a shift toward simulation-based assessment and the increasing use of multimodal data sources. Still, methodological heterogeneity, poor transparency, and a lack of validity and reliability prevent generalisability across tasks and contexts. AI-based assessment has potential in surgical education, with real-time adaptive assessment particularly interesting; however, standardised, validated methods are required to ensure reproducible measures of performance and ethical implementation in surgical simulation training.

## Introduction and background

Background

Surgical training increasingly employs simulation to enhance technical competence and patient safety within controlled, risk-free environments [1,2]. While early simulators facilitated repetitive practice, the absence of standardised assessments limited objective feedback and structured skill acquisition across training levels. Artificial intelligence (AI), defined as computer software capable of performing tasks that typically require human intelligence, has transformed training by automating performance measurement [3,4]. AI-based assessments analyse simulation data to produce reproducible and unbiased metrics that more effectively differentiate novices, intermediates, and experts compared to global rating scales [5,6]. This significantly reduces reliance on subjective instructor opinions, as AI provides scalable, real-time evaluations of surgical training proficiency [3,7]. These technologies enhance learning productivity, provide personalised feedback, and improve patient safety by requiring trainees to demonstrate competence before performing procedures. This paradigm shift underscores the critical role of digital technologies in developing reproducible, efficient, and measurable surgical education programs. Automated surgical skill assessment primarily relies on motion tracking, gesture recognition, and haptic feedback as key methods for performance evaluation. Motion tracking records tool or hand movements, measuring orientation, path length, smoothness, and speed during surgical procedures [8,9]. Gesture recognition segments procedures into predefined elements, facilitating error identification, workflow analysis, and expert norm comparison [3,10]. Robotic platforms, such as the da Vinci system, generate standardised kinematic machine learning datasets for outcome-prediction tasks [10,11]. Haptic feedback captures applied forces, tissue interaction, and tool handling, providing insights into quality and patient risk [12,13]. The integration of gesture, motion, and haptic data enhances predictive accuracy and enables real-time feedback, which is often absent in instructor-based evaluations. Collectively, these modalities offer reproducible, objective, and personalised training platforms, reducing patient risk and surpassing traditional subjective evaluation systems. Computer vision and deep learning technologies further enhance automated evaluation by improving the accuracy and scalability of surgical performance assessments. Computer vision analyses surgical videos for instrument detection, gesture recognition, and error detection without the need for specialised hardware, allowing for extensive applications [5,14]. Deep learning algorithms, trained on annotated data, achieve high accuracy in workflow segmentation and skill classification [13,15]. Validation employs construct validity for expertise discrimination, criterion validity for expert rating comparisons, and predictive validity for training outcome associations [14,16]. Challenges include sensor variability, heterogeneous datasets, limitations in external generalisability, and algorithmic bias [4,7]. Nonetheless, AI, motion tracking, haptic feedback, and computer vision facilitate reproducible, standardised assessment frameworks. These innovations enhance learning effectiveness, increase patient safety, and establish evidence-based training that better prepares surgeons for actual clinical environments. Previous systematic reviews have examined AI in surgical education and virtual reality (VR)-based training, often focusing narrowly on single modalities. This review broadens the scope by encompassing studies from 2010 to 2025, integrating bibliometric trends, and synthesising findings across multiple modalities. This comprehensive perspective provides a more complete understanding of automated and AI-based assessment in surgical education.

Rationale

AI-based and automated assessment methods currently represent a diverse and dispersed array. Researchers employ motion data, force sensors, haptic feedback, and computer vision; however, the evidence is dispersed across numerous studies with varying validation frameworks. Educators and clinicians necessitate a comprehensive synthesis of these methods to facilitate their adoption. This systematic review seeks to address this need by evaluating the evidence on the validity, accuracy, and reliability of AI- and automation-based performance assessments. By systematically reviewing the literature, this study elucidates the methodological strengths and weaknesses, enabling the identification of the most suitable data modalities and assessment strategies for integration into routine surgical training. A systematic synthesis is also imperative due to ongoing challenges with reproducibility and generalisability. Current studies frequently rely on single-institution datasets and proprietary simulators, which constrain external validity demands of annotation and inconsistencies in reporting further complicate comparisons across algorithms and platforms. Educators risk adopting tools without clear guidance or robust evidence of effectiveness or fairness. This review will underscore key limitations by examining prevalent biases, reporting gaps, and failure modes. The findings will serve as a foundation for standardising testing, informing future development, and advising accreditation bodies seeking to incorporate automated testing into competency-based surgical education.

Objectives and review questions

The goal of this review is to conduct a systematic review of the use of AI and machine learning methods in evaluating performance in surgical simulation settings. Specifically, this review will identify the types of AI or automated methods currently used to assess performance and evaluate their accuracy against expert evaluation or other validated criteria. It will also review the validity evidence, including evidence for construct validity, criterion validity, and predictive validity, and the reliability of the methods for performance evaluation. Lastly, this review will evaluate which data modalities, surgical procedure types, and simulator types have the best and most accurate assessment of performance, identify common biases present in the literature, highlight common failure modes in practice, and suggest areas for development and future research in AI-based assessment in surgical education.

## Review

Methods

This review followed the Preferred Reporting Items for Systematic Reviews and Meta-Analyses (PRISMA) 2020 statement. An attempt was made to register this review with the International Prospective Register of Systematic Reviews (PROSPERO); however, formal registration could not be completed due to a persistent technical error within the platform's automated classification system. To maintain high standards of transparency and reproducibility in the absence of a registered protocol, the review was conducted in strict accordance with the PRISMA 2020 statement. Furthermore, inclusion and exclusion criteria were established a priori using the Population, Intervention, Comparison, Outcomes, and Study (PICOS) framework, and the selection process, comprising independent dual-reviewer screening and the use of a third-party arbitrator, is documented in the provided PRISMA 2020 flow diagram. Data extraction was performed using a standardised template to ensure the comparability and synthesis of results. The process was designed to ensure transparent reporting, reproducibility, and minimal bias. At least two reviewers (AK and OT) independently screened all records, with disagreements resolved by discussion or arbitration by a third reviewer (MA).

Inclusion Criteria

The population of interest (P) involves participants engaged in simulated surgical operations; this could include medical students, residents, fellows, or attending surgeons. Studies are eligible regardless of the simulation modality, including benchtop, VR, augmented reality (AR), robotic, or cadaveric simulators. The intervention (I) is defined by automated or AI-driven assessment methods, including computer vision, kinematic assessment, force or torque sensing, eye tracking, or natural language processing (NLP) applied to system log files that produce performance scores or determine skill levels. The comparator (C) involves human raters who utilise established tools (e.g., Objective Structured Assessment of Technical Skills (OSATS) and Global Evaluative Assessment of Laparoscopic Skills (GOALS)), established thresholds, or predefined levels of expertise. The outcomes (O) can include the ability to discriminate skill levels, correlation with expert ratings, performance, or classification of skill, i.e., area under the curve (AUC), accuracy, or F1 score, and prediction score metrics, i.e., mean absolute error (MAE) and root mean square error (RMSE). Reliability measures, i.e., test-retest reliability or inter-rater reliability, as well as efficiency measures related to speed or resource allocation, are also examined. The study designs (S) could include experimental, observational, or validation studies, with the use of simulation in the experiment. Only English-language studies published from database inception to the search date are included, while non-English studies identified during the search are recorded for transparency.

Exclusion Criteria

The exclusion criteria for this review were established to ensure that only studies focused directly on AI-enabled performance assessment within surgical simulation were included. In particular, narrative reviews, systematic reviews, meta-analyses, editorials, and opinion pieces were not included in the review. Algorithm or methodology papers that did not constitute evaluation within simulated surgical tasks were reviewed. If a study is based solely on real-world surgical video data or intraoperative data without a simulation aspect, it was also removed from consideration. Any study, including conference abstracts, that does not include enough methodological detail or enough outcome data was also removed from the review. Non-English full-text articles were also excluded if translation was not possible.

Search Strategy

The search strategy was developed using prior reviews and adapted for this study's eligibility criteria. Four databases were searched from inception up to September 30, 2025: PubMed, Embase, Scopus, and IEEE Xplore Digital Database. Controlled vocabulary and free-text terms were combined with Boolean operators and truncation to capture variations in terminology. Filters restricted results to English-language publications. The final strategy included terms for surgical simulation, AI-based assessment, and performance evaluation. Reference lists of the included studies and reviews were hand-searched. 

Search String

The search string for each database is detailed as follows: for PubMed, ("surgical simulation" OR "surgical training" OR "surgical education" OR "surgical skills" OR "skills training" OR "surgical simulator" OR "training simulator" OR "virtual reality" OR "virtual reality training" OR "virtual reality surgery" OR "augmented reality" OR "mixed reality" OR "XR simulation" OR "laparoscopic simulation" OR "laparoscopy training" OR "robotic surgery" OR "robotic training" OR "benchtop simulator" OR "task trainer" OR "cadaveric simulation" OR "skills laboratory" OR "skills lab" OR "procedural training") AND ("artificial intelligence" OR Al OR "machine learning" OR "deep learning" OR "neural network" OR "computer vision" OR "video analysis" OR "automation" OR "automated assessment" OR "automated scoring" OR "automated evaluation" OR "automated feedback" OR automated performance OR motion tracking" OR "kinematics" OR "kinematic analysis" OR "sensor based" OR "wearable sensors" OR haptics OR "force feedback" OR "patter recognition" OR "data driven" OR "algorithmic assessment" OR "predictive model") AND ("skill assessment" OR "skills assessment" OR "performance assessment" OR "performance evaluation" OR "technical skills" OR "technical performance" OR "competency assessment" OR "competence evaluation" OR "objective assessment" OR "construct validity" OR "criterion validity" OR "predictive validity" OR "expert rating" OR "expert evaluation" OR OSATS OR GOALS OR "global rating scale" OR "surgical performance"); for Scopus, ("surgical simulation" OR "surgical simulator" OR "surgical training simulator" OR "laparoscopic simulation" OR "laparoscopic trainer" OR "virtual reality surgery" OR "VR surgery" OR "robotic surgery training" OR "robotic simulation" OR "benchtop simulator" OR "task trainer") AND ("artificial intelligence" OR "machine learning" OR "deep learning" OR "computer vision" OR "automated assessment" OR "automated performance" OR "automated evaluation" OR "motion tracking" OR kinematics OR "kinematic analysis" OR "force sensing" OR haptics); for Embase, ("surgical simulation" or "surgical simulator" or "surgical training simulator" or "laparoscopic simulation" or "laparoscopic trainer" or "virtual reality surgery" or "VR surgery" or "robotic surgery training" or "robotic simulation" or benchtop simulator" or "task trainer") and ("artificial intelligence" or "machine learning" or "deep learning" or "computer vision" or "automated assessment" or "automated performance" or "automated evaluation" or "motion tracking" or kinematics or "kinematic analysis or force sensing" or haptics)).mp. mp=title, abstract, heading word, drug trade name, original title, device manufacturer, drug manufacturer, device trade name, keyword heading word, floating subheading word, candidate term word; and for IEEE Xplore, "surgical simulation" OR "surgical simulator" OR "surgical training simulator" OR "laparoscopic simulation" OR "laparoscopic trainer" OR "virtual reality surgery" OR "VR surgery" OR "robotic surgery training" OR "robotic simulation" OR "benchtop simulator" OR "task trainer" ) AND ( "artificial intelligence" OR "machine Iearning" OR "deep Iearning" OR "computer vision" OR "automated assessment" OR "automated performance" OR "automated evaluation" OR.

Study Selection

All the acquired references were exported into Zotero reference management software (Corporation for Digital Scholarship, Vienna, Virginia, United States), where duplicate detection was automatically done, supplemented by manual verification to cross-check for accuracy. Two reviewers (AK and OT) independently screened titles and abstracts, with potentially relevant studies assessed in full text. Disagreements were resolved by consensus or third reviewer adjudication (MA). Excluded studies at the full-text stage were documented with the reasons for exclusion. The whole selection process is summarised in a PRISMA 2020 flow diagram.

Data Extraction

Two reviewers independently extracted data using a standardised template. Extracted information included study identifiers, design, participants, simulation environment, data modalities, AI/automated methods, comparators, outcomes, and validation techniques. Reliability, computational requirements, feedback mechanisms, and key findings were also recorded. Discrepancies were resolved by discussion or adjudication. Final data were compiled into structured tables to support transparency, comparability, and synthesis.

Results

Search Results

**Figure 1:**
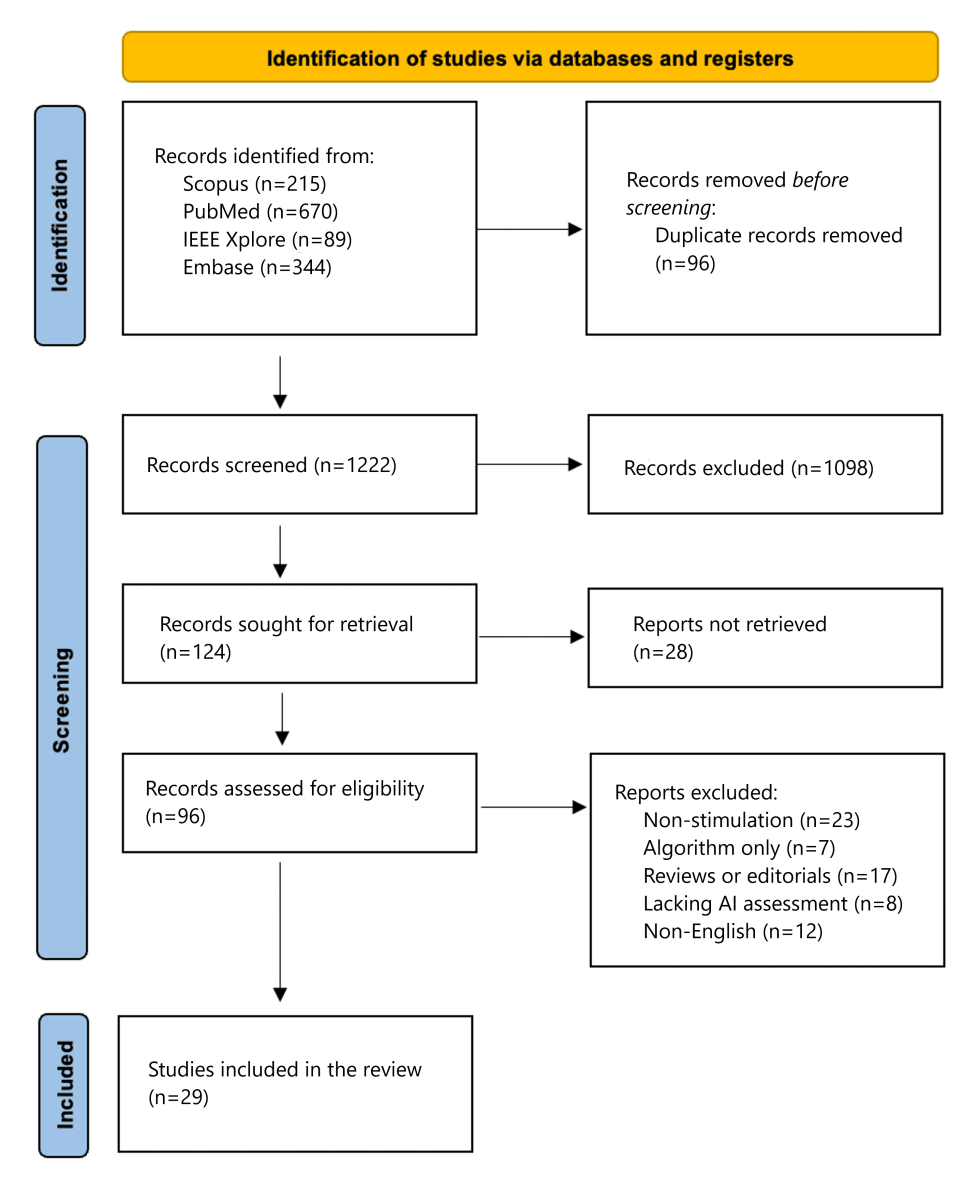
PRISMA flowchart showing the study selection process PRISMA: Preferred Reporting Items for Systematic Reviews and Meta-Analyses; AI: artificial intelligence

Database searches identified 1,318 records: PubMed (n = 670), Embase (n = 344), Scopus (n = 215), and IEEE Xplore (n = 89). After deduplication, 1,222 unique records remained. Title and abstract screening excluded studies that did not meet population, intervention, or outcome criteria. Ninety-six full texts were reviewed in detail, with disagreements resolved by consensus or third reviewer adjudication. Of these, 29 studies met all inclusion criteria and were included in the final analysis. The most common reasons for exclusion at the full-text stage were lack of automated or AI-based assessment, absence of simulation, or missing outcome data. The selection process is summarised in the PRISMA 2020 flow diagram (Figure 1) [17]. 

Study Characteristics

A summary of the key characteristics and performance metrics of the included studies is provided in Table 1, while the detailed data extraction is available in the Appendices.

**Table 1:** Summary of the study characteristics and key AI performance metrics AI: artificial intelligence; AUC: area under the curve; SVM: support vector machine; VR: virtual reality; ML: machine learning; AR: augmented reality; LSTM: Long Short-Term Memory; MAE: mean absolute error; DNN: deep neural network; KCF: kernelized correlation filter; CNN: convolutional neural network; RAS: robotic-assisted surgery; BABA: bilateral axillo-breast approach; OSATS: Objective Structured Assessment of Technical Skills; GEARS: Global Evaluative Assessment of Robotic Skills; FCNN: fully convolutional neural network; LH-OSATS: Laparoscopic Hysterectomy-Objective Structured Assessment of Technical Skills; ICC: interclass correlation coefficient; GOALS: Global Evaluative Assessment of Laparoscopic Skills; SHAP: Shapley Additive Explanations; FLS: Fundamentals of Laparoscopic Surgery; ANN: artificial neural network; IOL: intraocular lens

Study (author, year)	Surgical task/simulator	Data modality and AI method	Key outcomes (accuracy/validity)
Sugiyama et al., 2025 [18]	End-to-side anastomosis (artificial vessels)	Video; ResNet-50+YOLOv2	AUC 0.85-1.00; high gesture recognition
Singh et al., 2023 [19]	Craniotomy drilling (3D-printed bone)	Force myography; Naive Bayes/SVM	90% accuracy in skill classification
Prevezanou et al., 2024 [20]	VR laparoscopic (LAP Mentor™)	VR metrics; 7 ML models+LSTM	97-99% accuracy; strong predictive validity
Pisla et al., 2025 [21]	AR pancreatic surgery (ATHENA robot)	Visual+haptic; EfficientNetV2B0	MAE 0.0244N; precise force prediction
Pan et al., 2023 [22]	da Vinci suturing (JIGSAWS)	2D video; KCF+ResNet	84.8-92.04% accuracy; construct validity
Nakajima et al., 2025 [23]	Laparoscopic colorectal surgery	Intraoperative video; EfficientNetB7	0.91 accuracy; moderate correlation (0.54) with manual scores
Nakajima et al., 2024 [24]	Laparoscopic sigmoidectomy	Intraoperative video; EfficientNetB7	0.86 accuracy in phase recognition
Moglia et al., 2022 [25]	Robotic-assisted surgery (dV-Trainer)	Simulator metrics; ensemble DNN	R² 0.79-0.96; predicted proficiency curves
Mirchi et al., 2020 [26]	Brain tumour resection (NeuroVR)	VR kinematic+force; linear SVM	92% accuracy; 100% sensitivity for experts
Luongo et al., 2021 [27]	Vesicourethral anastomosis (live robot)	Video; CNN+LSTM	AUC 0.88; identified suturing gestures
Li et al., 2024 [28]	Clutch-based hand motion (RAS)	Video; skin color+rule-based	Differentiated novices from experts (p<0.05)
Lee et al., 2020 [29]	BABA robotic thyroidectomy	Surgical video; mask R-CNN	83% accuracy; high OSATS/GEARS correlation
Khanfar et al., 2025 [30]	Peg transfer (Box trainer)	Eye-tracking; K-means++ clustering	Clustering successfully differentiated proficiency
Kasa et al., 2022 [31]	One-handed knot-tying	Image+kinematic; multimodal ResNet	Construct validity confirmed (p=0.0038)
Karlik et al., 2021 [32]	Brain tumour resection (NeuroVR)	Kinematic; hybrid FCNN	Confirmed construct validity via expert grouping
Jokinen et al., 2020 [33]	Laparoscopic hysterectomy (LAP Mentor)	Simulator metrics (non-AI study)	Construct validity of LH-OSATS (p=0.01)
Ismail Fawaz et al., 2019 [34]	da Vinci JIGSAWS (suturing/knot)	Kinematic; FCNN	Construct validity via interpretability heatmaps
Fazlollahi et al., 2023 [35]	NeuroVR tumour resection	Kinematics+force; SVM	High internal consistency vs. expert benchmarks
Fazlollahi et al., 2022 [36]	NeuroVR tumour resection	Kinematics+force; SVM+LSTM	ICC 0.84; high agreement with expert ratings
Ismail Fawaz et al., 2018 [37]	da Vinci JIGSAWS	Kinematic; 1D CNN	Accuracy benchmarked against state-of-the-art
Funke et al., 2019 [38]	da Vinci JIGSAWS	Video; I3D+Temporal Segment Net	High accuracy, recall, and F1 scores
Ebina et al., 2025 [39]	Box trainer (porcine dissection)	Optical MoCap; PCA-SVM	GOALS correlation 0.86-0.91; provided SHAP feedback
Brown and Kuchenbecker 2023 [40]	Robotic peg transfer	Accelerometers+force; Regression ML	Evaluated automated GEARS feedback impact
Bogar et al., 2024 [41]	VR vs. FLS box peg transfer	Video+VR data; YOLOv8	95% agreement between AI and expert raters
Belmar et al., 2023 [42]	Box trainer (bean drop/peg transfer)	Video; YOLOv4+U-Net	93.02% agreement for peg transfer
Atroshchenko et al., 2025 [43]	Robotic cardiac tasks (porcine)	Video; CNN+LSTM	93% accuracy for action recognition
Anh et al., 2020 [44]	da Vinci JIGSAWS	Kinematic; SVM/CNN	92.75-96.84% accuracy; CNN performed best
Alonso-Silverio et al., 2018 [45]	Box trainer (MISTELS tasks)	Video (webcam); ANN	93.94% accuracy; AUC 0.97
Akada et al., 2025 [46]	Intrascleral IOL fixation (eye)	Wearable strain sensors; LightGBM	86.9-87.9% accuracy for microsurgery

Risk of Bias Assessment

**Figure 2:**
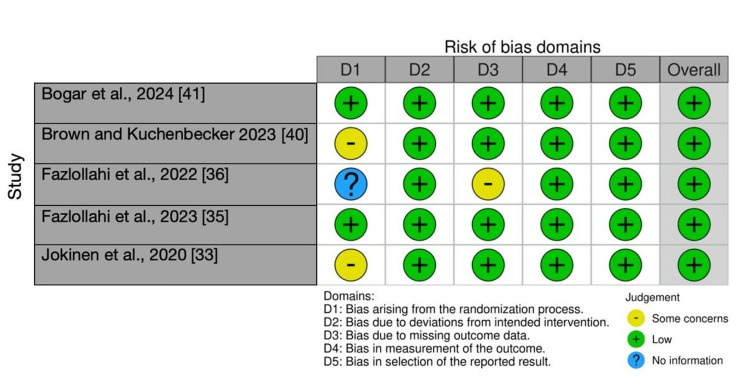
Traffic light plot of critical appraisal of the studies

**Figure 3:**
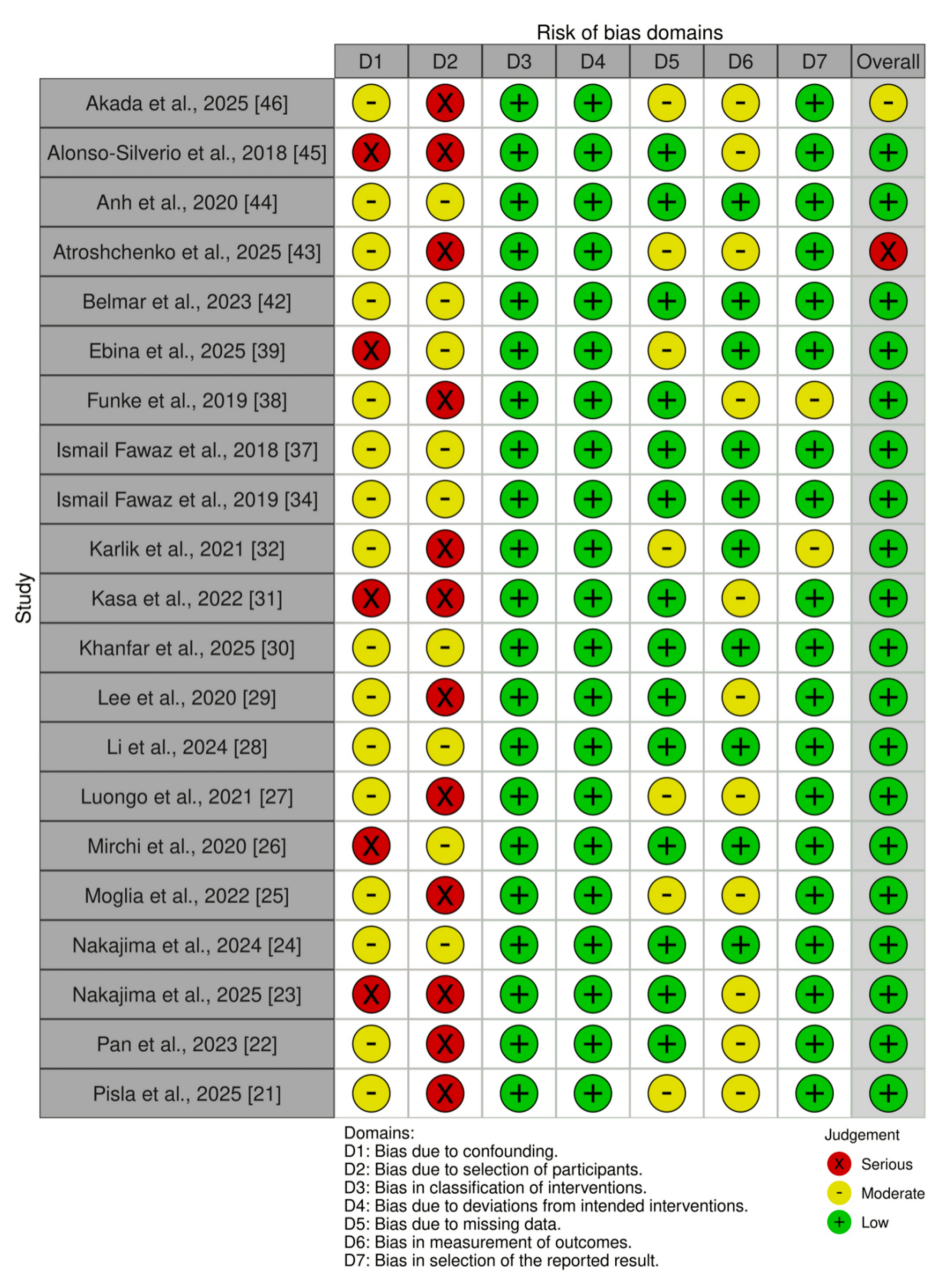
Traffic light plot of critical appraisal of the studies

The risk of bias in randomised controlled trials (RCTs) was evaluated using the Cochrane RoB 2 tool [47], while non-randomised studies were assessed using the ROBINS-I tool [48]. The majority of RCTs demonstrated a low risk of bias, particularly concerning outcome measurement and missing data. Two trials consistently exhibited low risk across all domains. Other trials raised concerns, primarily regarding randomisation and incomplete reporting. Non-randomised studies displayed greater variability, with risk levels ranging from low to serious. The most prevalent issues included confounding, selection bias, and unclear classification of interventions. Retrospective designs tended to exhibit higher risk scores, whereas prospective studies with standardised protocols generally received lower ratings. Bias was frequently associated with small cohort sizes and a lack of multicentre validation, highlighting methodological limitations across the included studies. A visual summary of domain-level ratings is provided in Figures 2-3, with comprehensive details available in Tables 2-3.

**Table 2:** Risk of bias assessment (RoB 2) for randomised controlled trials

Study	D1	D2	D3	D4	D5	Overall
Bogar et al., 2024 [41]	Low	Low	Low	Low	Low	Low
Brown and Kuchenbecker 2023 [40]	Some concerns	Low	Low	Low	Low	Low
Fazlollahi et al., 2022 [36]	No information	Low	Some concerns	Low	Low	Low
Fazlollahi et al., 2023 [35]	Low	Low	Low	Low	Low	Low
Jokinen et al., 2020 [33]	Some concerns	Low	Low	Low	Low	Low

**Table 3:** Risk of bias assessment (ROBINS-I) for non-randomised studies

Study	D1	D2	D3	D4	D5	D6	D7	Overall
Akada et al., 2025 [46]	Moderate	Serious	Low	Low	Moderate	Moderate	Low	Moderate
Alonso-Silverio et al., 2018 [45]	Serious	Serious	Low	Low	Low	Moderate	Low	Low
Anh et al., 2020 [44]	Moderate	Moderate	Low	Low	Low	Low	Low	Low
Atroshchenko et al., 2025 [43]	Moderate	Serious	Low	Low	Moderate	Moderate	Low	Serious
Belmar et al., 2023 [42]	Moderate	Moderate	Low	Low	Low	Low	Low	Low
Ebina et al., 2025 [39]	Serious	Moderate	Low	Low	Moderate	Low	Low	Low
Funke et al., 2019 [38]	Moderate	Serious	Low	Low	Low	Moderate	Moderate	Low
Ismail Fawaz et al., 2018 [37]	Moderate	Moderate	Low	Low	Low	Low	Low	Low
Ismail Fawaz et al., 2019 [34]	Moderate	Moderate	Low	Low	Low	Low	Low	Low
Karlik et al., 2021 [32]	Moderate	Serious	Low	Low	Moderate	Low	Moderate	Low
Kasa et al., 2022 [31]	Serious	Serious	Low	Low	Low	Moderate	Low	Low
Khanfar et al., 2025 [30]	Moderate	Moderate	Low	Low	Low	Low	Low	Low
Lee et al., 2020 [29]	Moderate	Serious	Low	Low	Low	Moderate	Low	Low
Li et al., 2024 [28]	Moderate	Moderate	Low	Low	Low	Low	Low	Low
Luongo et al., 2021 [27]	Moderate	Serious	Low	Low	Moderate	Moderate	Low	Low
Mirchi et al., 2020 [26]	Serious	Moderate	Low	Low	Low	Low	Low	Low
Moglia et al., 2022 [25]	Moderate	Serious	Low	Low	Moderate	Moderate	Low	Low
Nakajima et al., 2024 [24]	Moderate	Moderate	Low	Low	Low	Low	Low	Low
Nakajima et al., 2025 [23]	Serious	Serious	Low	Low	Low	Moderate	Low	Low
Pan et al., 2023 [22]	Moderate	Serious	Low	Low	Low	Moderate	Low	Low
Pisla et al., 2025 [21]	Moderate	Serious	Low	Low	Moderate	Moderate	Low	Low

Bibliometric Analysis

**Figure 4:**
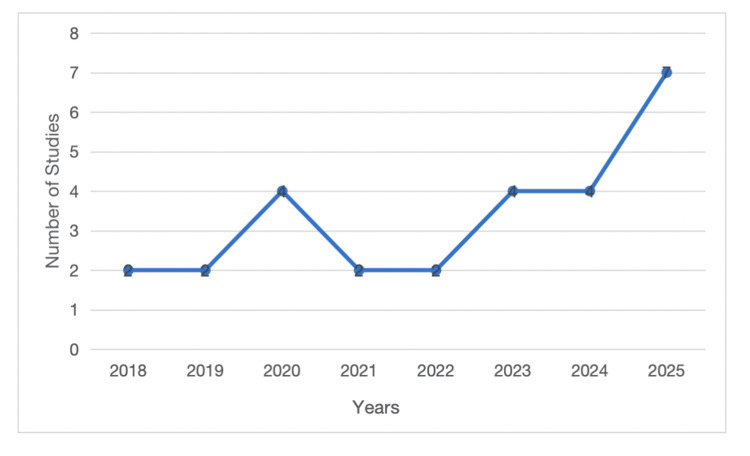
Number of studies over the years

Year of publication: The studies covered illustrate an increasing trend of publications on AI-based surgical assessment over the past few years. Most studies were published in 2025, as interest in research and technology in this field continues to grow [18,21,23,30,39,43,46]. Four studies were reported in 2024 [20,24,28,41] and 2023 [19,22,35,42], indicating ongoing development and testing activities. Early years, such as 2022 [25,31] and 2021 [27,32], included fewer studies, each with two, reflecting the relative freshness of this study area. This trend continued in 2020, with four studies [26,29,33,44], and 2019 [34,38] and 2018 [37,45], each with two studies, which is the initial phase of AI and automated methods in simulation-based testing. Generally, the spread indicates an upward trend, suggesting accelerated adoption and exploration of AI tools in surgical training. The trend also identifies the growing importance of AI-driven approaches to increasing training effectiveness, streamlining examination, and enabling competency-based progression. The spread of studies across the years is provided in Figure 4.

**Figure 5:**
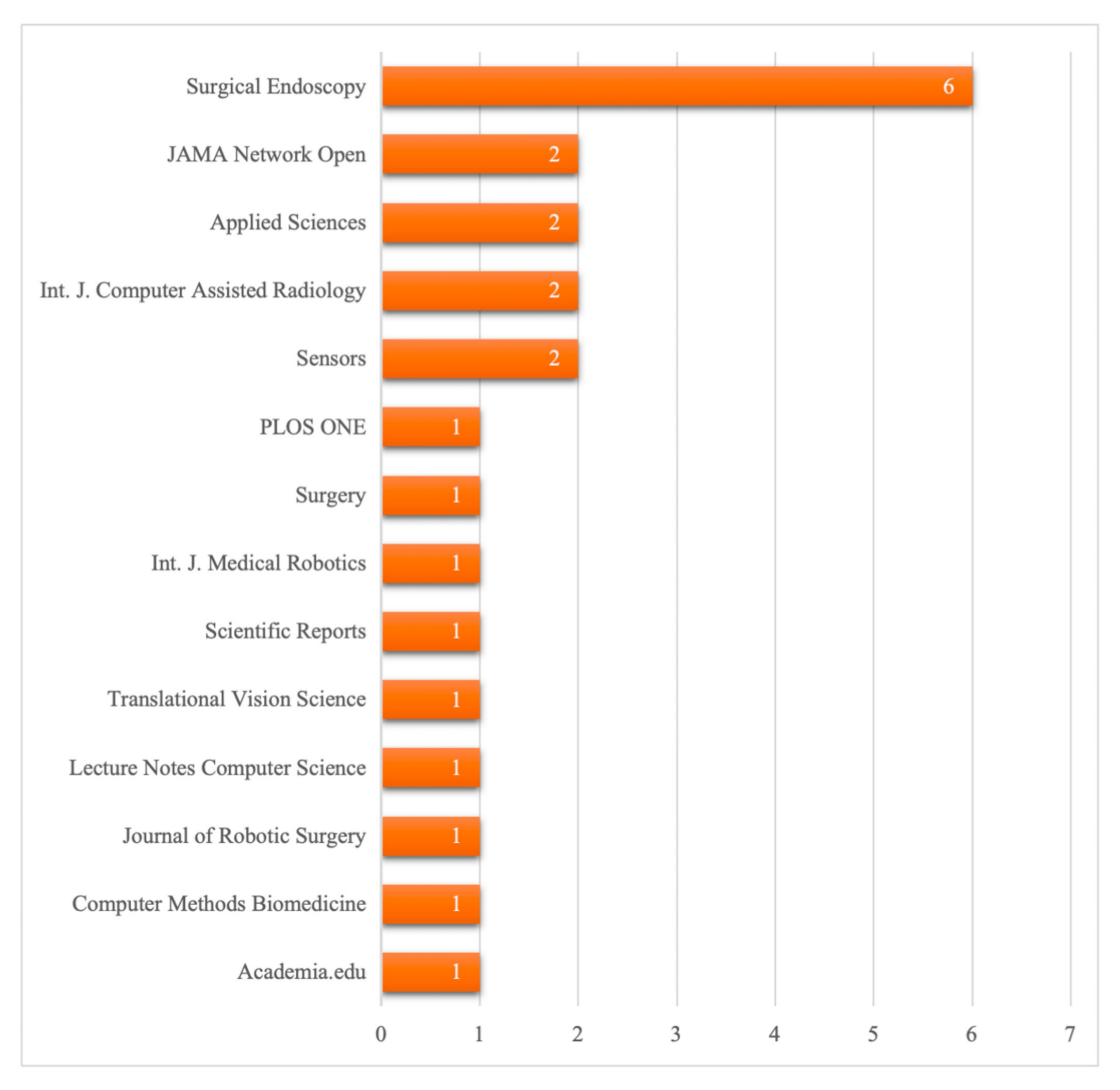
Number of studies by source of publication

Sources of publication: The articles were distributed in different journals, with Surgical Endoscopy leading the pack with six articles. JAMA Network Open and Applied Sciences trailed with two, demonstrating clinical and engineering perspectives. The International Journal of Computer Assisted Radiology and Surgery also had two, pointing to the relevance of computation. Other journals, like PLOS One, Sensors, Surgery, The International Journal of Medical Robotics + Computer Assisted Surgery, Scientific Reports, Translational Vision Science & Technology, Lecture Notes in Computer Science, Journal of Robotic Surgery, Computer Methods and Programs in Biomedicine, and Academia.edu, yielded one study each, showing diversity in publication outlets and interdisciplinarity. Overall, the distribution indicates that research on AI-based surgical assessment appears in both surgical and technology journals, suggesting a cross-disciplinary field. The geographical distribution of sources is illustrated in Figure 5.

**Figure 6:**
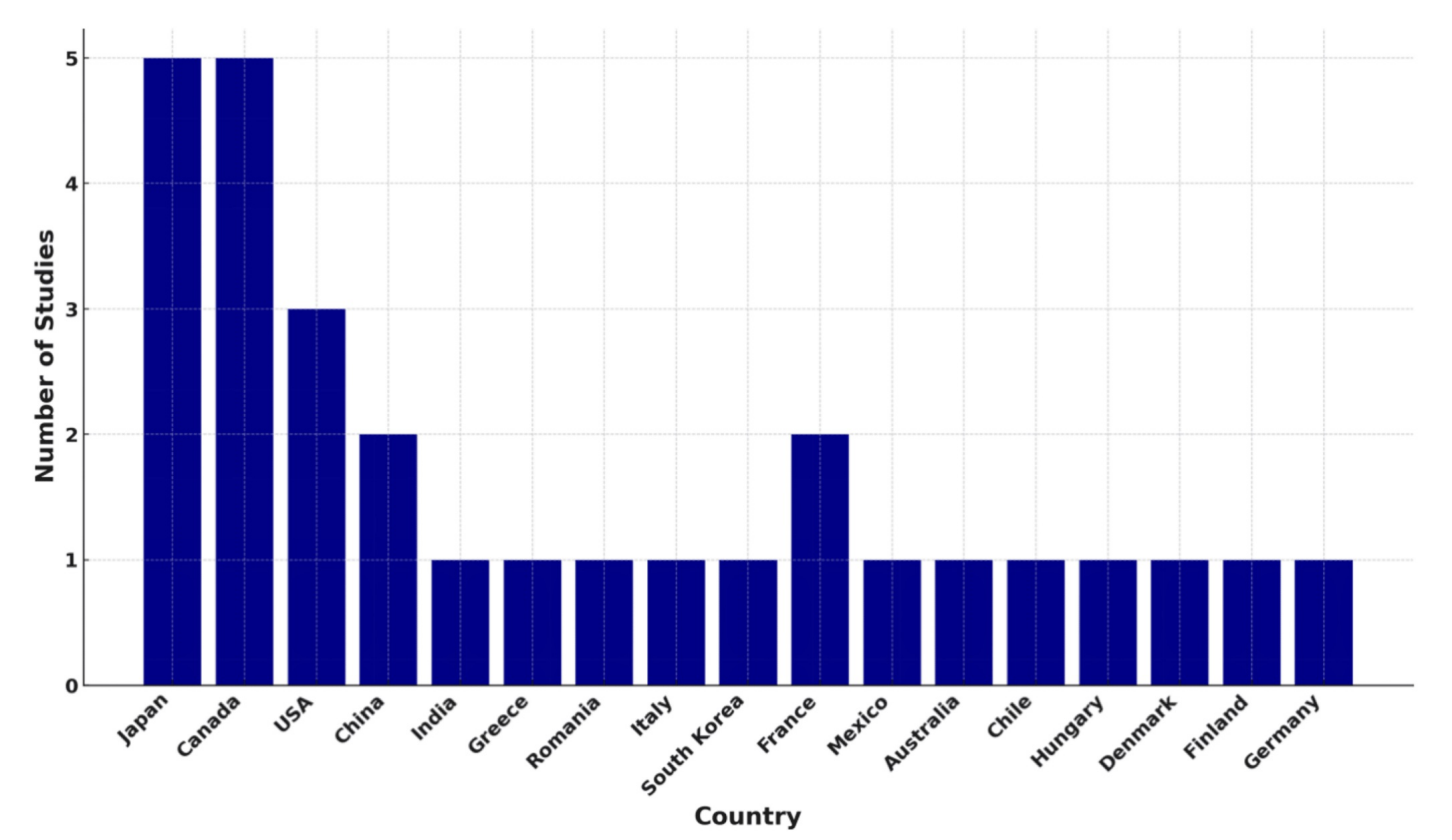
Number of studies by country

Geographical distribution (country-wise): The studies participating are from diverse countries, reflecting global interest in AI-based surgical assessment. The leaders are Japan [18,23,24,39,46] and Canada [26,31,32,35,36], with five studies each, indicating vigorous research activity in technological development and clinical application. The United States follows with three [27,30,40], followed by China with two [22,28], indicating growing East Asian activity. Several other nations, including India [19], Greece [20], Romania [21], Italy [25], South Korea [29], France [34,37], Mexico [45], Australia [44], Chile [42], Hungary [41], Denmark [43], Finland [33], and Germany [38], have one or two studies, indicating widespread but more scattered research activities. This spread suggests cross-national coordination and adoption of AI-supported surgical training and assessment. Across geography, there is a potential for varying approaches, infrastructure, and access to simulators. Overall, the studies show an equitable mix: the wealthiest countries are innovation leaders, and the rest contribute unique perspectives and pilot studies. The studies are classified by country in Figure 6.

**Figure 7:**
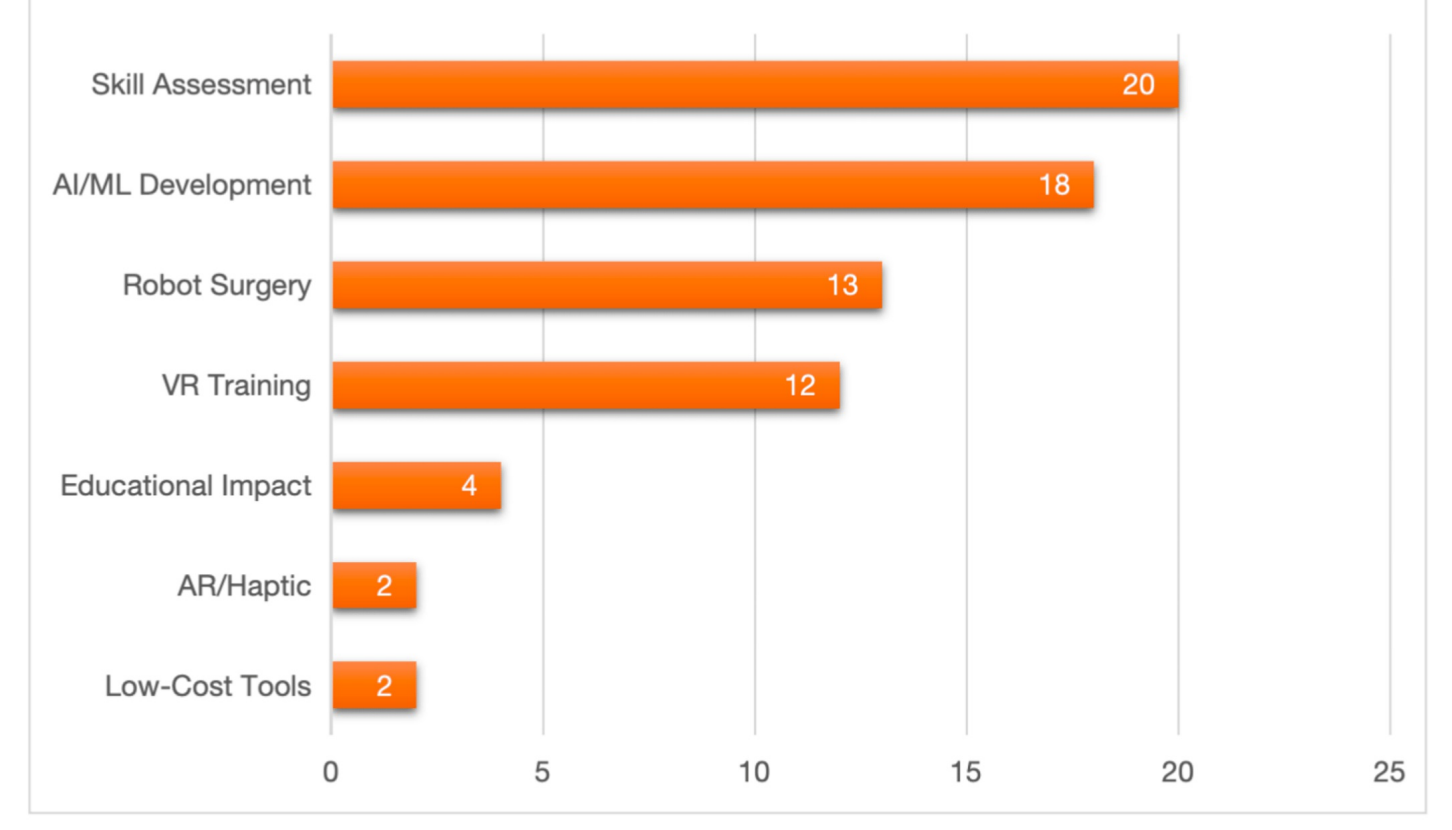
Number of studies by research areas AI: artificial intelligence; ML: machine learning; VR: virtual reality; AR: augmented reality

Research areas: The research questions cover many topics that align with the multidisciplinary nature of surgical training and assessment. Robot-assisted surgery and equivalent procedures were the focus of 13 studies, highlighting the importance of advanced operative technology [20-22,25,27-29,34,37,38,40,43,44]. Training on VR and simulators was the focus of 12 studies [20,25,26,33-37,39,41,42,45], attesting to interest in interactive and controlled settings. Machine learning algorithm construction and AI ranked highest at 18 studies [18-29,31,32,35-38], indicating a strong emphasis on computerised competence assessment, predictive modelling, and creation of feedback. Skill assessment metrics such as OSATS, Global Evaluative Assessment of Robotic Skills (GEARS), Global Rating Scale (GRS), Endoscopic Surgical Skill Qualification System (ESSQS), GOALS, and kinematic or force measurements were used in 20 studies [19,20,22-29,31-34,36,37,39,40,42,45], underscoring the centrality of objective assessment in surgical training. Two studies [21,30] focused on AR and haptic feedback. Through these, there could be an enhanced user experience while maintaining performance accuracy. Cost-effective, hardware-driven, or feasibility-directed tools were used in two studies that emphasised real-world strategies to improve accessibility [19,45]. In four studies, the influence of educational and curricula was evaluated, showing how AI and simulation can impact teaching methods and the delivery of learning outcomes [33,36,40,41]. The grouping of studies by research area is shown in Figure 7.

Findings

AI or Automated Methods Used

Algorithm type: Algorithmic techniques used to evaluate surgical skill range from traditional machine learning and deep learning to hybrid models. Sugiyama et al. [18] used a ResNet-50 combined with YOLOv2 to detect and classify gestures during end-to-side anastomosis surgery and demonstrated strong performance in recognition and prediction. Singh et al. [19] compared several traditional machine learning models, including Naive Bayes, linear discriminant analysis (LDA), support vector machines (SVM), and decision trees, for skill classification in craniotomy drilling, and Naive Bayes achieved the highest accuracy of 90%. Prevezanou et al. [20] applied seven machine learning algorithms in conjunction with Long Short-Term Memory (LSTM) networks to assess VR laparoscopic simulation tasks and demonstrated predictive performance for improvement. Pisla et al. [21] employed an EfficientNetV2B0 convolutional neural network (CNN) to perform AR-based simulations of minimally invasive pancreatic surgery and managed to map visual and haptic inputs to force prediction. These studies collectively demonstrate that deep convolutional networks and LSTM-based architectures dominate the state of the art in AI-based surgical evaluation. At the same time, conventional machine learning methods remain viable in small datasets or lower-dimensional feature spaces. Algorithm choice typically varies with data type, problem complexity, and the necessity of real-time performance, emphasising an individualised strategy for different surgical simulations.

Feature/data modality: Data modality selection and feature extraction are crucial for correctly evaluating AI-based performance. Pan et al. [22] utilised 2D endoscopic video of da Vinci suturing procedures, allowing for gesture and motion analysis. Nakajima et al. [23] combined intraoperative video with red-green-blue (RGB) and optical flow features, capturing visual appearance and motion dynamics during laparoscopic colorectal surgery. Moglia et al. [25] employed simulator-generated metrics, including task completion time, errors, and motion smoothness, as input to deep neural networks to predict student proficiency in robotic-assisted surgery. Mirchi et al. [26] combined VR kinematic and force data from subpial brain tumour resection simulation so that machine learning models could detect expert and novice skill levels well. Across these studies, data modalities included video and motion sensors, haptic feedback, and force measurement, with multimodal combinations improving the performance of AI. High-resolution visual data integration and precise kinematic or force measurements allow models to recognise fine detail changes in skill, motion economy, and tool use. Modality selection is task-dependent; multimodal approaches optimally serve surgical simulations with sophisticated motor control or tactile assessment.

Task type solved by AI: Surgical simulation application of AI is task type-dependent, reflecting clinical significance and technical complexity. Luongo et al. [27] discussed vesicourethral anastomosis suturing in actual robot-assisted surgery using two-stream CNNs with LSTM layers for gesture recognition. Li et al. [28] investigated a clutch-based hand motion task using computer vision to track fine movements, distinguishing expert versus novice. Lee et al. [29] evaluated instrument movement and predicted OSATS and GEARS scores during bilateral axillo-breast approach (BABA) robotic thyroidectomy using intraoperative video. Khanfar et al. [30] evaluated peg transfer tasks in adult and child box trainers for Fundamentals of Laparoscopic Surgery (FLS), with eye-tracking and clustering algorithms to classify skill levels based on fixations, saccades, and task completion measures. Such studies validate that AI can excel at procedure subtasks and global performance evaluation. Algorithm choice is affected by the task choice, feature extraction methods, and validation strategy. CNNs and temporal models support recurring or high-accuracy operations like suturing and clutch actions, while attention-based, coordination-based, or hand-eye integration-based tasks rely on clustering, eye tracking, or multimodal analysis.

Real-time capability: Real-time feedback is increasingly significant for surgical training and assessment. Kasa et al. [31] demonstrated that multimodal ResNet-based methods can provide real-time skill ratings by combining kinematic and visual features to predict GRS automatically. Karlik et al. [32] used fully convolutional neural networks (FCNNs) to provide near-instant estimates of surgical ability during NeuroVR simulations for direct trainee or instructor feedback. Atroshchenko et al. [43] used CNN-LSTM architectures in AR-supported atrial closure procedures, providing accurate real-time augmented feedback, though skill measurement accuracy was not consistently high. Akada et al. [46] used high-precision wearable sensors with LightGBM models trained on fresh features, enabling near-real-time inference of finger movement metrics for ocular surgery tasks. Real-time performance in all these applications is a function of model complexity, feature dimensionality, and computational resources like graphics processing units (GPUs) or specially optimised central processing units (CPUs). Successful deployment requires high frame-rate video, low-latency sensor data, and high-performance inference pipelines. Real-time AI feedback enhances skills acquisition by providing prompt corrective instruction, highlighting errors, and supporting expert-level motion, allowing for more adaptive and customised surgical training.

Expert or Benchmark vs. Accuracy

Overall metrics of accuracy: Overall accuracy is a primary measure of AI performance relative to expert or gold-standard benchmarks. Sugiyama et al. [18] reported AUC values of 0.85-1.00 for identifying gestures during end-to-side anastomosis, indicating near-perfect discrimination for some tasks. Singh et al. [19] achieved 90% classification accuracy with Naive Bayes for craniotomy drilling skill levels, demonstrating that non-deep or conventional machine learning is as effective as deep learning, provided that appropriate features are selected. Prevezanou et al. [20] attained 97-99% accuracy using seven machine learning models combined with LSTM, where the strength of temporal models in predicting skill acquisition from VR laparoscopic simulation is highlighted. Pan et al. [22] reported task classification accuracies ranging from 84.8% to 92.04% for da Vinci suturing tasks, demonstrating that CNN-based vision models can replace traditional sensors without compromising precision. Across these studies, maximum aggregate accuracy demonstrates the potential of AI-guided surgical skill evaluation, with minimal differences across data complexity, task specificity, and model type. Accuracy, however, must be interpreted alongside task- and expert-level measurements to determine clinical utility.

Expert/benchmark correlation: Expert correlation of evaluation defines the criterion validity of AI models in surgery evaluation. Nakajima et al. [23] reported an overall accuracy of 0.91 and a moderate correlation of 0.542 between EfficientNetB7 outputs and manual dissection efficiency scores in laparoscopic colorectal surgery, with conformation to expert opinion. Nakajima et al. [24] achieved an overall accuracy of 0.86 in phase identification, with a Pearson correlation of r=0.415 with expert ESSQS rankings, confirming the AI's capacity to identify phase-specific performance nuances. Mirchi et al. [26] achieved 92% accuracy, 100% sensitivity, and 82% specificity in differentiating expert/novice performance of VR subpial brain tumour resections, with high agreement against ground-truth skill annotations. Lee et al. [29] demonstrated a prediction accuracy of 83% and an AUC of 0.86 for BABA robotic thyroidectomy performance, further confirming that expert-rated OSATS and GEARS ratings correlate with deep learning-based tracking. These studies show that AI models can achieve agreement with expert ratings, serving as an effective surrogate for human ratings and reducing the time and effort required for manual scoring.

Task-specific metrics: Task-specific metrics provide precise data on AI performance beyond broad classification. Pisla et al. [21] measured a MAE of 0.0244 N and a maximum deviation of 0.045 N in AR-guided minimally invasive pancreatic surgery, illustrating precise force prediction for real-time application. Moglia et al. [25] reported R² coefficients ranging from 0.79 to 0.96 for attempts and time on VR robotic-assisted surgery tasks, with significant predictive value for proficiency curves. Li et al. [28] quantified clutch-based hand motion tasks and achieved clutch accuracy percentages sufficient to differentiate novices from experts, illustrating the sensitivity of AI to small-scale dexterity traits. Brown and Kuchenbecker [40] measured force integrals, trial lengths, and tool acceleration during peg transfer tasks, taking objective measurements of motor efficiency. These task-specific measures reflect AI models' capacity to identify subtle motor performance features, supplementing total accuracy metrics. They affirm the value of AI in the exact evaluation of surgical skill, particularly for mundane, quantifiable aspects such as force delivery, motion smoothness, and task timing, where conventional observation may be imprecise.

Validity and Reliability

Construct validity: Construct validity tests whether the AI models are measuring the intended skill constructs. Nakajima et al. [23] grouped surgeons into high, intermediate, and low skill groups and compared dissection efficiency ratios using AI, with higher ratios in experts and a fair correlation with manual scoring. Nakajima et al. [24] applied phase recognition to laparoscopic sigmoidectomy videos and observed shorter phase durations and fewer transitions between experts, confirming that AI accurately captures performance variation across skill levels. Kasa et al. [31] also confirmed multimodal ResNet-50+ResNet-18-LSTM outputs against OSATS GRS using analysis of variance (ANOVA), finding notable differences between novices and experts (p=0.0038). Fazlollahi et al. [36] combined Implicit Competency Evaluation in Multimodal Simulation (ICEMS) deep learning scores and OSATS performance for preclinical medical students and demonstrated that AI-based assessment followed improvement patterns accorded construct expectations. These studies establish that AI models validly assess underlying surgical competencies, quantifying procedural capability objectively. Construct validity is particularly evident when models are diverse across skill levels and align with conventional proficiency scales, supporting their use in formative and summative assessment in simulation and live-surgery settings [44].

Criterion validity: Criterion validity tests AI performance against a gold-standard expert rating. Mirchi et al. [26] demonstrated that a linear SVM could distinguish novice from expert performance with 92% accuracy, demonstrating convergence with expert standards. To determine criterion validity, Lee et al. [29] showed a strong correlation between predicted outcomes and OSATS/GEARS scores in BABA robotic thyroidectomy. Sugiyama et al. [18] compared ResNet-50+YOLOv2 outputs with Stanford scale ratings by three trained professionals, showing high agreement in skill ratings at the gesture level. Singh et al. [19] compared Naive Bayes, LDA, SVM, and decision tree predictions with Likert-scale ratings and experience levels for craniotomy drilling. They showed high conformity with expert-derived assessment measures. These studies demonstrate that AI algorithms can match human benchmarks, achieving high levels of agreement across various surgical tasks and modalities. Criterion validity is critical to clinical and training adoption, as AI-based assessments must be assured that they align with widely accepted performance standards and are reliably interpretable as equivalent to expert judgment, promoting trust in automated assessment systems.

Reliability/reproducibility: Reliability measures how consistent AI measurements are under replicated conditions. Nakajima et al. [23] did not explicitly test for repeatability across the same surgeons, but observed trends within overall groups as a sign of consistent efficiency-ratio output. Nakajima et al. [24] did not test reliability but used cross-validation to generalise to new cases and assess reproducibility across the dataset. Kasa et al. [31] reported interclass correlation coefficients (ICC) of 0.70-0.83 for expert scores and test-retest ICC 0.49-0.88 for multimodal model outputs, reflecting moderate to high reliability. Fazlollahi et al. [35] demonstrated higher internal consistency in SVM-based AI feedback among medical residents during simulated neurosurgery procedures, with consistent performance across trial runs. These studies underscore that AI can provide dependable skill measures, while outright repeatability testing is scant across most studies. High reliability and established construct and criterion-related evidence increase the validity of AI-driven surgical assessment instruments, particularly for repeated formative measurement or longitudinal skill tracking [44].

Data Modalities, Tasks, and Simulator Types

Simulator type: The selection of the simulator plays a pivotal role in AI-based surgical skill assessment. Prevezanou et al. [20] used the LAP Mentor VR system for laparoscopic tasks, using abundant VR performance information to train LSTM-based AI models with 97-99% accuracy for predicting the progress stage. Moglia et al. [25] used the dV-Trainer VR simulator for robotic-assisted surgery tasks, and ensemble deep neural network (DNN) models estimated proficiency curves with R² of 0.79-0.96, which validates VR's capability for systematic measurement of skills. Khanfar et al. [30] combined FLS box and custom-made pediatric trainers, quantifying eye-tracking and NASA-Task Load Index (TLX) measures to differentiate between novice and expert performance using K-means++ clustering. Bogar et al. [41] compared a Meta Quest 2 VR system to normal FLS box trainers for peg transfer tasks and found equivalent training effectiveness, with AI grading significantly reducing test time. These studies indicate that VR and physical box trainers provide adequate fidelity for AI testing, with the added benefit of increased sensor integration and the potential for real-time feedback in VR. The choice of simulator influences data richness, task variety, and AI performance, with multimodality treatments pushing the frontiers of reliability and usability in skill assessment and learning [33].

Task type/complexity: Task selection impacts the performance and usability of the AI model. Sugiyama et al. [18] investigated end-to-side vascular anastomosis with artificial vessels using fine motor precision, which was well represented by ResNet-50+YOLOv2. Singh et al. [19] compared craniotomy drilling on 3D-printed bone matrices using Naive Bayes and SVM models, correctly classifying novices, intermediates, and experts based on force myography data. Pisla et al. [21] considered minimally invasive pancreatic surgery with an AR-robotic trainer that integrated visual and haptic feedback to predict force deviations in real time using EfficientNetV2B0. Pan et al. [22] tested robotic suturing on da Vinci systems, combining 2D endoscopic video with kernelized correlation filter (KCF)+ResNet to accurately classify practice skill levels. These tests demonstrate that task complexity directly affects feature extraction and model performance. Multimodal data capture benefits tasks requiring fine motor control and multi-step sequencing and encourages high-quality AI assessment. Task-specific modelling ensures that AI outputs reflect important distinctions between skills rather than superficial performance measures.

Effectiveness of data modality: Data modality selection plays an essential role in the performance of AI prediction. Ebina et al. [39] used 120 Hz optical motion capture to capture instrument kinematics during dissection and suturing, which correlated strongly with GOALS scores and provided interpretable Shapley Additive Explanation (SHAP)-based explanations. Ismail Fawaz et al. [37] employed JIGSAWS 1D kinematic signals in a CNN using Class Activation Maps and achieved near-perfect suturing and needle passing classification. Funke et al. [38] demonstrated that video-based I3D+temporal segment networks achieved 95-100% accuracy, demonstrating the capability of sole video inputs to compare to kinematic data. Alonso-Silverio et al. [45] used a simple webcam video in a box trainer, feeding it into a neural network to estimate skill with 93.9% accuracy and an AUC of 0.97, proving that low-cost video can still yield reliable AI assessment. Generally, the experiments indicate that although high-fidelity motion capture enhances model accuracy, video-based and kinematic modalities are sufficient to achieve precise skill classification when supported by robust AI architectures. Multimodal integration also enhances sensitivity and interpretability.

Biases, Failure Modes, and Reporting Gaps

Sample size and distribution: The number and distribution of participants exert a significant effect on AI generalisability. Nakajima et al. [23] analysed 766 laparoscopic colorectal procedures across three skill levels, providing moderate statistical power to compare AI predictions with manual dissection effectiveness. Nakajima et al. [24] expanded this dataset to 1,572 videos, enabling strong phase identification and skill stratification, although cross-surgeon variability remained an issue. Singh et al. [19] validated 22 mixed-expertise neurosurgeons, which, while optimal for pilot validation, limits generalisability to larger groups or heterogeneous surgical contexts. Fazlollahi et al. [35] validated 46 preclinical medical students with high internal consistency, highlighting that homogeneous, small samples can potentially overestimate AI performance metrics. Collectively, these studies emphasise the importance of well-balanced and adequately sized cohorts for robust training and validation of AI. Uneven group sizes or underrepresentation of skill levels can introduce bias into predictive modelling and amplify the algorithm's performance, particularly when applied in real-world clinical training or assessment. Sample heterogeneity is required to permit generalisability of AI-based skill assessment across a wide population.

Missing/incomplete reporting: A lack of reporting can limit reproducibility and transparency in AI-enabled surgical assessment. Atroshchenko et al. [43] reported limited reliability in skill assessment (SA) labelling due to single-annotator data, which affects the generalisability of AR-based feedback. Brown and Kuchenbecker [40] noted that there was no basis for automatic GEARS feedback, reducing the interpretability of accurate force and motion modelling. Lee et al. [29] did not provide code, constraining independent model verification of deep learning models for BABA robotic thyroidectomy. Moglia et al. [25] did not test repeatability in the same surgeon, leaving predictive consistency uncertain. These exclusions, combined, indicate the persisting problem in surgery AI research: a lack of complete transparency into training procedures, hyperparameters, and validation protocols. Without complete method reporting, models are not appropriately externally valid, limiting reproducibility and their use in educational or clinical settings. Emphasising the systematic recording of data preprocessing, model selection, and evaluation strategies is critical to ensure findings are readily verifiable and applicable beyond the original study context.

Task-specific failure/accuracy gaps: AI performance may also be task- and situation-specific. Ebina et al. [39] reported 56.9-67.3% classification accuracies in surgical action recognition, which was moderate predictive reliability with high-quality optical motion capture. Belmar et al. [42] indicated lower agreement for the bean drop task (79.69%) compared with peg transfer (93.02%), suggesting that task difficulty influences object detection and skill evaluation. Brown and Kuchenbecker [40] indicated that AI feedback did not significantly influence trainee performance, demonstrating that sound measures don't always equate to skills acquisition. Akada et al. [46] recorded 87.9% and 86.9% accuracy for two tasks (CP1, CP2), demonstrating that high-precision sensor systems still face limitations in distinguishing nuanced skill differences. These studies indicate that task-related factors, such as motion variability, task complexity, and sensor fidelity, impact AI performance. Determining failure modes and accuracy gaps is vital for effective improvement and informed deployment of AI-augmented surgical training. Task-specific performance measurement confirms limitations and responds to them appropriately.

Discussion

Synthesis of Results

Results were collated, including AI and automated approaches, algorithm types, task complexity, simulators, data modalities, performance measures, validity, reliability, and bias analysis. Bibliometric trends were then analysed year by year, journal by journal, country by country, and by research topic.

Principal Findings

AI and automated evaluation methodologies effectively quantify surgical proficiency across various simulation environments. Techniques such as CNNs, recursive models, and ensemble methods are employed to differentiate skill levels, predict outcomes, and deliver immediate feedback with minimal human intervention [49]. Multimodal data, encompassing kinematics, video, and force data, surpasses repeated single-modality predictions. Established tools, including OSATS, GEARS, and expert scores, exhibit a high correlation with AI predictions, despite task variability [49]. Robotic suturing, laparoscopic skills, and neurosurgical simulation are particularly pertinent, with repetitive, structured tasks demonstrating the greatest consistency in performance. Sample size influences accuracy, with larger multicentre groups enhancing generalisability, whereas smaller cohorts may risk overestimating performance [50]. Real-time evaluation is feasible for video, haptic, and AR modalities, although computational requirements vary based on algorithmic complexity. AI systems diminish the need for instructor oversight, facilitating continuous objective evaluation and immediate performance feedback [51]. The high accuracy, reliability, and feasibility of AI-enhanced assessment across diverse environments suggest its potential to expedite skill development, provide actionable feedback, and complement traditional evaluation methods [52]. The integration of these tools enables efficient, reproducible, and standardised skill assessment, emphasising areas where human judgment is essential.

Interpretation

AI demonstrates optimal performance in tasks characterised by well-defined, repetitive steps with consistent motion patterns, such as peg transfer, suturing, and robotic motion [27,38,41]. However, its efficacy diminishes in procedures of high complexity, those requiring nuanced decision-making, interpretation of variable anatomical structures, or flexible reasoning [4,24]. The integration of video, kinematics, and force data enhances model accuracy, enabling the discrimination of subtle skills and the detection of errors [31]. External validation using expert criteria confirms the reliability of AI models and quantifies discrepancies between algorithmic outputs and human assessments, thereby mitigating bias and overfitting [16,39]. Variability in performance is influenced by task complexity, participant skill level, and sensor precision. It indicates that AI is most effective for quantifiable tasks and less reliable for those requiring interpretive judgment [20,31]. Providing transparent, explainable outputs and aligning with expert human opinion facilitates adoption and trust in AI systems [26]. Standardised preprocessing, feature selection, and cross-validation techniques are employed to prevent model overfitting and enhance reproducibility. While AI offers objective performance metrics, continuous monitoring, and predictive feedback, human judgment remains essential for clinical reasoning and context-dependent decision-making [11,36]. Recognising the strengths and limitations of AI allows for its careful application, maximising benefits while avoiding unnecessary overuse in activities that demand subtle decision-making and expertise [4,35].

Strengths and Limitations

Integrated analysis elucidates AI performance in surgical tasks, simulative modalities, and algorithm types. The synthesis of 29 diverse studies offers comprehensive insights into the application of machine learning and neural networks for assessing surgical skills. Notable strengths include the systematic classification of simulators, data modalities, task types, and algorithmic strategies, facilitating cross-study comparisons. Methodological rigour is demonstrated through the use of benchmark metrics, systematic extraction, and objective performance evaluation [17]. A further strength of this review is its extensive scope: unlike previous systematic reviews that focused solely on VR or single assessment methods, this review spans 2010-2025, incorporates bibliometric mapping, and synthesises across multiple modalities. This breadth provides a more comprehensive and current overview than prior work, elucidating temporal and thematic shifts within the literature. Limitations arise from heterogeneity in findings, partial reporting of model parameters, limited reproducibility, real-time capability, and varying sample sizes [53]. The reliance on small sample sizes and single-centre datasets presents a significant barrier to the broader clinical adoption of these technologies. While studies like those by Nakajima et al. [24] utilised larger datasets (n=1,572), many others, such as Singh et al. [19] (n=22) and Fazlollahi et al. [35] (n=46), relied on smaller, more homogeneous cohorts. Such limited sample sizes risk overestimating AI performance metrics, as models trained on narrow datasets may lack the diversity needed to account for the wide variability in surgical technique and anatomical presentation found in the general population. Consequently, the high accuracy rates reported in several studies may not hold when these algorithms are applied to heterogeneous surgical contexts or different training populations. Furthermore, there is a critical need for external validation and prospective multicentre trials to translate these experimental findings into real-world clinical training. Current evidence is heavily skewed toward single-institution studies, which frequently lack cross-institutional testing to confirm that performance remains stable across different surgical environments and platforms. Without such validation, the reliability of AI-based assessments remains confined to the specific conditions of the original study. Transitioning from pilot validation to prospective, multicentre frameworks is essential to establish the predictive validity of these tools, ensuring they truly correlate with long-term clinical outcomes and skill retention in diverse learning cohorts. Small cohorts risk inflating performance estimates, while multicentre datasets would enhance generalisability but remain scarce. Many included studies lacked reproducibility frameworks or external validation, limiting reliability across institutions. Differences in preprocessing, validation, and feature selection further complicate algorithmic comparisons. Task-related variability and inconsistent reporting standards make it unclear whether performance remains stable across settings. These methodological weaknesses reflect broader challenges in surgical simulation research, including issues of reproducibility, reliance on single-centre data, and the use of narrow cohorts. Despite these constraints, this synthesis outlines the successful applications of AI, highlights modalities and tasks that maximise accuracy, and clarifies factors influencing reliability. Identifying such strengths and limitations provides practical guidance for implementing AI-assisted assessment and designing robust, reproducible systems for surgical training and simulation. The consequences of incomplete reporting further hinder progress in this field. A significant portion of the literature lacks transparency regarding model hyperparameters, validation protocols, and source code. For instance, the absence of shared code in studies such as Lee et al. [29] prevents independent verification and limits the ability of other researchers to build upon established models. This lack of transparency directly undermines the reproducibility of AI research in surgical simulation. To foster trust among stakeholders, future research must prioritise exhaustive validation and transparency protocols, including the systematic recording of data preprocessing and model selection, which are vital for ensuring that findings are verifiable and applicable beyond their original study context.

Implications for Stakeholders

Surgical trainees and medical students: New AI and computer-assisted grading tools are changing the face of surgical education by providing ongoing, real-time, and objective feedback on technical competencies. Such systems allow trainees to identify both their strengths and weaknesses, allowing them to develop more quickly than is possible with only instructor evaluations [40,41]. Real-time measurement of motion patterns, vectors of force, and completion of the procedure allows the trainee to practice independently and also become exposed repeatedly to different simulated scenarios, allowing for the attainment of mastery of the skill [22,42]. Feedback that includes a social comparison of the trainee to other trainees or expert performance can provide direction on learning objectives and improve motivation toward self-improvement [51]. These systems lessen dependence on subjective assessments, foster independence, promote confidence, prioritise early error detection and correction, and generally increase preparedness for the live procedure. Furthermore, it is important to balance objective quantitative feedback with subjective qualitative feedback so as to minimise reliance on the numerical score. Traditionally, simulation with AI-assisted guidance adds richness to personalised development of skills, increases efficiency in learning skills, and provides adjuncts to traditional mentorship without removing it [11,26].

Surgical educators and faculty supervisors: Computer assessment tools offer instructors a method of performance calibration that is consistent, inexpensive, and calibrated with minimal variability in feedback for participants [54]. This affords educators greater bandwidth for engaging in opportunities to teach higher-order constructs such as advanced decision-making and clinical reasoning, while AI is responsible for oversight of lower-order technical skills. Accurately determining skill deficits and objective metrics of development will further support competency-based advancement with focused remediation and a structured method for mentoring. Continuous assessment and data aggregation provide opportunities to assess trends across trainees for constructing a curriculum and improving methodology [55]. The use of AI for feedback generates a capacity for educators to minimise the time commitment and effort associated with formal assessment and generate standardised measures of progress across an entire cohort or class [52]. However, educators must ensure that scores are well-attended to context and that the assessment is not mechanically or automata-responded. The use of AI teachers can support more efficient teaching and more precise assessments of limited students in transfer and provide an evidence-based means for mentoring.

Hospital and simulation centre administrators: AI-backed assessment provides actionable data for resourcing, evaluating training, and program assessment, and, as it shifts evaluation from an observation-based to an AI-supported model, it reduces the need for faculty supervisory support [40,42]. Faculty members can scale to larger cohorts without additional faculty, while performance indicators provide quantifiable data for simulator scheduling, module purchases, and technology investments [14,42]. Standardised testing provides evidence for program accreditation, quality assurance, and competency milestone reporting. Data-driven planning leverages data to allocate resources (employees, equipment, and operational planning) more efficiently and effectively (thus, it costs less). Administrators now need to consider the budgets required to maintain hardware, software, and technical support staff. The addition of AI-based assessments increases program efficacy, quality training, and institutional reputation.

AI-based surgical assessment tool developers: Data reveal which input modalities, task types, and model structures yield valid performance estimations. Developers may focus on kinematics, video, force, and multimodal fusion while optimising algorithms for accuracy, efficiency, and interpretability [5,22]. Awareness of common biases and the risks of overfitting underscores the importance of diverse training sets and robust validation. Alignment with expert standards ensures that outputs are comprehensible and reliable. Open design and real-time feedback capabilities enhance usability and adoption. Adhering to these guidelines facilitates the development of functional, effective, and safe AI tools for surgical education.

Future Research Directions

Future directions for research should focus on testing the reliability of AI assessment tools across a range of institutions and learners to enable generalisability. Using different data types or task categories may strengthen predictive validity. Investigating longer-term learning outcomes, skill retention, and the influence of AI-based formative feedback on clinical performance is critical in future research. Comparative studies can demonstrate the contribution of AI-based assessment methods against traditional methods. Future research should thoroughly investigate ethical issues, transparency, user-friendliness, cost-effectiveness, and scalability. Furthermore, working collaboratively with institutional administrators, educators, and software developers will be a vital component of implementation strategies.

Proposition 1: standardised multimodal data framework: Develop a standardised structure for multimodal data fusion in surgical assessment, including kinematics, video, and force data. By utilising a single dataset format, uniform feature extraction processes can be achieved, instrument comparability improved, and validity strengthened by countering bias caused by a narrow or skewed dataset. The structure should establish minimum standards for data quality, data annotation, and data interoperability to foster collaborative efforts among institutions. Standardisation is designed to aid rapid development, validity testing, and adoption of AI assessment tools that generate reliable, reproducible, and applicable educational and learner-centred outputs.

Proposition 2: real-time adaptive feedback mechanisms: Develop adaptive AI feedback platforms that provide real-time, context-specific feedback within simulated environments. Feedback should be tailored to the trainee's level, the task's complexity, and the learner's objectives. Adaptive systems can target specific errors, suggest opportunities for adjustment, and dynamically recalibrate the difficulty level to enhance overall learning efficiency. Integrating intuitive visualisation, comprehensible scoring, and analytically actionable feedback will assist in participant engagement and understanding. The learner's journey around mastery learning, self-development, and competence building is enhanced, as these evidence-based methods recognise the principle of AI support of instructor guidance, not the replacement of some aspect of teaching or feedback.

Proposition 3: exhaustive validation and transparency protocols: Validation and transparency protocols establish rigorous validation protocols for AI scoring tools, including cross-institutional testing, diverse trainee populations, and benchmarking against human expert scorers. To foster user trust, prioritise transparency of model architecture, feature identification, and scoring algorithms. Documentation should clearly outline system limitations, potential biases, and expected accuracy ranges. Continuous monitoring and recalibration with real-world use data will ensure long-term reliability and relevance. Formulating these standards ensures that AI-driven assessment is valid, ethically responsible, and practical for implementation in surgical education programs.

## Conclusions

AI and automated assessment tools have considerable promise for improving surgical simulation training by providing accurate technical skill assessments, improved task efficiency, and procedural correctness. When multimodal inputs (e.g., motion kinematics, video, force) are used, objective tools provide even richer quantitative assessments that are more robust than human observation and can support individualised self-paced practice, competency-based progression, and evaluation against expert benchmarks. Educators benefit from consistent, standardised assessment, while administrators gain valuable data for evaluating the original allocation of resources and programs, and developers receive actionable information for evaluating sensor/algorithm design, implementation, and use. However, when implementing AI/automated assessments in practice, it is essential to address potential bias in the model and ensure that model explainability, validation, and contextual implementation issues are addressed. Where bias is addressed, AI/automated assessment provides a scalable, replicable addition to surgical training that has the potential to improve learning experiences, instructional efficiency, and preparation for clinical practice.

## Appendices

**Table 4:** Comprehensive data extraction and technical specifications of the included studies AI: artificial intelligence; AUC: area under the curve; AIIMS: All India Institute of Medical Sciences; FMG: force myography; LDA: linear discriminant analysis; SVM: support vector machine; VR: virtual reality; ML: machine learning; LSTM: Long Short-Term Memory; MAE: mean absolute error; AR: augmented reality; HD: high definition; CNN: convolutional neural network; GRS: Global Rating Scale; KCF: kernelized correlation filter; LOSO: leave-one-super-trial-out; ESSQS: Endoscopic Surgical Skill Qualification System; RGB: red-green-blue; RAS: robotic-assisted surgery; DNN: deep neural networks; GBRT: gradient boosted regression trees; RARP: robot-assisted radical prostatectomy; BABA: bilateral axillo-breast approach; OSATS: Objective Structured Assessment of Technical Skills; GEARS: Global Evaluative Assessment of Robotic Skills; RMSE: root mean square error; ICC: interclass correlation coefficient; FLS: Fundamentals of Laparoscopic Surgery; MSE: mean squared error; ANOVA: analysis of variance; FCNN: fully convolutional neural network; FCM+BP (MLP): Fuzzy C-Means+Back-Propagation (Multi-Layer Perceptron); VAS: Visual Analogue Scale; CAM: Class Activation Map; SOTA: state-of-the-art; RCT: randomised controlled trial; VOA: virtual operative assistant; ICEMS: Implicit Competency Evaluation in Multimodal Simulation; I3D: Inflated 3D ConvNet; TSN: temporal segment network; BD: bean drop; PT: peg transfer; ITA: internal thoracic artery; FPS: frames per second; BORIS: Behavioural Observation Research Interactive Software; TBS: task-based score; PCA: principal component analysis; ANN: artificial neural network; IOL: intraocular lens

Study ID/citation	Country/setting	Study design	Participants (population)	Simulator/task	Data modality	Assessment/ground truth	AI/automated method	Validation/comparator	Performance metrics	Validity evidence	Reliability/reproducibility	Feedback type	Computational/resource info	Key findings/notes
Sugiyama et al., 2025 [18]	Japan	Validation study	14 surgeons (PGY 1-28)	End-to-side anastomosis, artificial vessels	Video	Stanford scale, 3 expert raters	ResNet-50+YOLOv2	Expert ratings	CV-VA, ΔVA, path distance, jerk index	AUC: 0.85/0.96/1.00	α=0.88-0.97; IoU=0.93	Potential real-time	MATLAB	Combined models superior
Singh et al., 2023 [19]	India/AIIMS	Validation study	22 neurosurgeons (6 experts, 8 intermediates, 8 novices)	Craniotomy drilling, 3D-printed bone matrix	FMG	Likert scale feedback, experience level	Naive Bayes, LDA, SVM, decision tree	Expert evaluation	5 FMG features (mean, variance, length, freq, CoV)	90% accuracy (Naive Bayes)	Leave-one-out validation	Post-procedure	Arduino, MATLAB	FMG is effective for skill assessment
Prevezanou et al., 2024 [20]	Greece/hospital simulation centre	Experimental	23 medical students (no prior experience)	LAP Mentor™ (Tasks 5, 6, 7)	VR performance metrics	Progress stages (beginning/middle/end)	7 ML+LSTM	20-fold CV	Accuracy, MAE	Prior construct validity	20-fold CV	Progress and remaining trials	Python, scikit-learn, TensorFlow	97-99% accuracy; LSTM MAE 3.6-4.0
Pisla et al., 2025 [21]	Romania/Technical University of Cluj-Napoca	Development/validation	15 users: 3 senior surgeons, 4 specialists, 3 residents, 5 engineers	AR surgical trainer for minimally invasive pancreatic surgery (ATHENA robot)	HD visual data, haptic inputs	Force measurements (Robotiq FT 300 sensor)	EfficientNetV2B0 CNN	Unity simulation vs. HoloLens 2; known vs. predicted forces	MAE: 0.0244N; max deviation 0.045N; latency 65 ms	Not specified	Not specified	Haptic (Omega 7)+AR visual feedback	Intel i9 CPU; PyTorch, Unity, MATLAB	Real-time force prediction; strong usability and novelty; eliminates the need for physical sensors
Pan et al., 2023 [22]	China/academic	Experimental	8 surgeons (novice, intermediate, expert)	da Vinci robot/suturing (JIGSAWS)	2D endoscopic video	Self-proclaimed skill (practice hours), GRS	KCF (tracking)+ResNet (classification)	LOSO cross-validation, comparison to LSTM/CNN	Accuracy, precision, recall, F1 score	Construct (motion features), criterion (GRS)	5 runs per input, mean accuracy reported	Automated skill level output	3-5 s inference time, Python/Keras/TensorFlow, i5-10400F, 16GB RAM	92.04% (2-class), 84.80% (3-class); vision replaces sensors; real-time feasible
Nakajima et al., 2025 [23]	Japan/multicentre	Retrospective	766 cases, 3 skill groups (high, intermediate, low)	Laparoscopic colorectal surgery (sigmoidectomy/high anterior resection)	Intraoperative video	ESSQS scores, manual annotation of dissection efficiency	EfficientNetB7 (CNN) for RGB+optical flow, instrument recognition	Correlation with manual measurement, group comparison	Precision, recall, F1 score, overall accuracy (0.91)	Construct (skill groups), criterion (manual ratio)	Not explicitly tested for the same surgeon repeatability	Automated efficiency ratio output	Not specified	Efficient-dissection time ratio is higher in the high-skill group (p<0.05); moderate correlation (0.542) with manual
Nakajima et al., 2024 [24]	Japan/multicentre	Retrospective	1572 videos, 3 skill groups (expert, intermediate, novice)	Laparoscopic sigmoidectomy/high anterior resection	Intraoperative video	ESSQS scores, manual phase annotation	EfficientNetB7 (CNN) for phase recognition	Correlation with manual annotation, group comparison	Precision, recall, F1 score, overall accuracy (0.86)	Construct (skill groups), criterion (ESSQS)	Not tested for same-surgeon repeatability	Automated AI score output	Not specified	Phase times (I, II, IV) and transition counts were lower in experts; AI score correlated with ESSQS (r=0.415)
Moglia et al., 2022 [25]	Italy/single centre	Prospective	176 medical students (no prior experience)	dV-Trainer (VR)/5 RAS tasks	Simulator metrics	Proficiency (consecutive green metrics)	Ensemble DNN	Comparison vs. random forest, GBRT, SVM	Accuracy (R²) for time and attempts	Construct (proficiency curves)	Not explicitly tested	Predictive feedback on proficiency	Keras/TensorFlow, scikit-learn	DNN outperformed other models (R² 0.79-0.96); early prediction is possible
Mirchi et al., 2020 [26]	Canada/academic medical centre	Validation study	50 participants: 28 skilled (staff, fellows, senior residents), 22 novice (junior residents, medical students)	NeuroVR; subpial brain tumour resection with ultrasonic aspirator and bipolar	VR kinematic and force data	Expert vs. novice classification based on training level	Linear SVM	Leave-one-out cross-validation	Accuracy: 92%; sensitivity: 100%; specificity: 82%	High classification accuracy; confusion matrix provided	Code publicly available on GitHub	Automated visual and auditory feedback; stepwise mastery learning	MATLAB R2018b; publicly available code	SVM with 4 metrics effectively classifies skill level; transparent feedback system
Luongo et al., 2021 [27]	USA/academic urology centre	Model development and validation	Surgeons performing RARP; video data from prior study	Live robot-assisted surgery; vesicourethral anastomosis suturing	Video (RGB+optical flow)	Manual annotation by 2 independent raters	Two-stream CNN (AlexNet)+LSTM/convLSTM	80/20 train/test splits ( × 3)	Identification AUC: 0.88; classification AUC: 0.87; top 1 accuracy: 62%	High AUC; confusion matrix analysis	Code not shared; hyperparameters detailed	Not a feedback system; foundational for automated assessment	AlexNet; LSTM/convLSTM; 7.5M-14.4M parameters	Deep learning CV can identify and classify suturing gestures in real surgery
Li et al., 2024 [28]	China/multicentre training	Feasibility study	40 surgeons: 30 laparoscopic (study group), 10 robotic experts (expert group)	dv-Trainer, EDGE MP1000, Toumai MT1000, da Vinci Xi; clutch-based tasks	Video (hand motion)	Manual annotation by 5 RAS experts	Computer vision (skin-color model+rule-based classification)	Comparison between study vs. expert groups	Number of clutches; clutch accuracy (%)	Significant differences between groups; p<0.05	Algorithm details provided; manual validation	Not a feedback system; assessment only	MATLAB; skin-color model for hand tracking	Clutch metrics distinguish skill levels; the accuracy gap persists after training
Lee et al., 2020 [29]	South Korea/academic hospital	Retrospective validation study	Surgeons (students, residents, fellows, professors); BABA training model and thyroid cancer patients	da Vinci robots (S, Si, Xi); BABA robotic thyroidectomy	Video (surgical view)	OSATS and GEARS scores by 2 surgeons	Mask R-CNN+Deep SORT+ReID for instrument tracking	5-fold cross-validation; comparison with expert ratings	Tracking RMSE: 3.52 mm; AUC (5 mm): 0.86; prediction accuracy: 83%	High correlation with OSATS/GEARS; ICC 0.71-0.74	Code not shared; detailed methods provided	Not a feedback system; assessment only	Mask R-CNN; Deep SORT; ST-ReID; BOVW-ReID	Deep learning tracking enables automated skill assessment; economy of motion is the most crucial metric
Khanfar et al., 2025 [30]	USA/academic simulation centre	Experimental study	28 participants: 24 novices, 4 experts	FLS adult and custom pediatric box trainers; peg transfer task	Eye-tracking (Tobii Glasses 3), NASA-TLX	Expert vs. novice performance; completion time, errors	K-means++ clustering	Comparison of clusters vs. expert performance	Fixations, fixation duration, saccades, pupil diameter, NASA-TLX	Clustering differentiated proficiency levels: significant differences from experts	Detailed eye-tracking protocol; statistical validation	Mixed-reality visual feedback (HoloLens 2)	Tobii Pro Lab; SPSS for analysis	Eye-tracking metrics distinguish skill levels; the pediatric trainer showed shorter fixations; feedback did not significantly affect metrics
Kasa et al., 2022 [31]	Canada/academic	Observational	72 surgical trainees and surgeons	One-handed knot-tying on polypropylene tubing	Image, kinematic (Leap Motion)	OSATS GRS (4 domains) by 3 expert raters	Multimodal ResNet-50+ResNet-18-LSTM	Human raters, ablation models	MSE, ICC, R², Spearman ρ, accuracy	Construct validity via ANOVA (p=0.0038)	ICC(2,3): 0.70-0.83; test-retest ICC: 0.49-0.88	Not specified	Pre-trained ResNet-50, 1D ResNet-18, Bi-LSTM; 100 epochs fine-tuning	The multimodal model outperformed unimodal and matched/exceeded human raters on most domains
Karlik et al., 2021 [32]	Canada/academic	Observational	50 participants: 18 experts, 10 senior residents, 10 junior residents, 12 medical students	NeuroVR (brain tumour resection)	Kinematic, performance metrics	Expert-defined skill groups (4 levels)	Hybrid FCNN (4 variants: FCNN-1 to FCNN-4)	Cross-validation; comparison to prior ML models (k-NN, LDA, Naïve Bayes, SVM)	Classification accuracy, error rate	Construct validity via expert grouping	Not explicitly reported	Not specified	FCNN with FCM+BP (MLP); 500-1500 iterations	FCNN-3 and FCNN-4 achieved 100% accuracy; outperformed prior models (90% max)
Jokinen et al., 2020 [33]	Finland/academic hospital	RCT	20 gynaecology residents	LAP Mentor (laparoscopic hysterectomy module)	Simulator metrics, video recording	OSATS (GRS+LH-specific), VAS; expert assessment	Not AI-based; simulator training intervention	Control group (no procedural simulator training)	OSATS scores, VAS scores, operative time, blood loss, complications	Construct validity of the LH-OSATS form (p=0.01)	ICC: 0.59-0.80; Cronbach's alpha: 0.80	Not automated; expert feedback from video review	LAP Mentor VR simulator; 10 training sessions	Simulator training improved OSATS and VAS scores (p<0.05); no significant difference in operative time/complications
Ismail Fawaz et al., 2019 [34]	France/academic	Retrospective analysis	8 subjects (2 experts, 2 intermediates, 4 novices)	da Vinci Surgical System (JIGSAWS: suturing, needle passing, knot tying)	Kinematic data (76 variables)	Self-reported skill level (novice, intermediate, expert); OSATS scores	FCNN	LOSO; comparison to S-HMM, ApEn, Sax-Vsm, CNN	Accuracy (micro/macro), Spearman's ρ	Construct validity via CAM interpretability	Not explicitly reported	Interpretable CAM-based heatmaps	1D CNN with grouped convolutions; Glorot init; Adam optimizer	SOTA accuracy (100% suturing/needle passing); CAM provides explainable feedback
Fazlollahi et al., 2023 [35]	Canada (Montreal)	Cohort study (secondary analysis of RCT)	46 medical students (preclinical), 14 expert neurosurgeons	NeuroVR (CAE Healthcare); simulated subpial tumour resection	54 variables at 50 Hz (e.g., position, force)	Expert benchmarks (mean performance of 14 neurosurgeons)	SVM-selected 4 learning objectives; VOA intelligent tutoring system	Control group (no feedback); expert benchmarks	270 metrics (safety, movement, efficiency)	Comparison to expert benchmarks; blinded expert OSATS ratings	High internal consistency; no loss to follow-up	Post-hoc audiovisual feedback on 4 AI-selected metrics	MATLAB, SPSS; SVM model; 50 Hz data logging	AI feedback improved safety but reduced efficiency and dominant hand speed; unintended learning effects were observed
Fazlollahi et al., 2022 [36]	Canada (Montreal)	RCT (instructor-blinded)	70 medical students (preclinical, years 0-2)	NeuroVR (CAE Healthcare); subpial tumour resection	54 variables at 50 Hz	Expert benchmarks; blinded OSATS; ICEMS deep learning score	VOA (SVM-based tutoring system); ICEMS (LSTM network)	Remote expert instruction; no feedback control	ICEMS expertise score; OSATS; 4 VOA metrics; 16 ICEMS metrics	OSATS (α=0.82); ICC=0.84; comparison to expert benchmarks	High internal consistency; no loss to follow-up	Post-hoc audiovisual (VOA); synchronous verbal (expert)	MATLAB; SPSS; SVM; LSTM	VOA outperformed expert instruction in ICEMS scores; equivalent OSATS and affective outcomes
Ismail Fawaz et al., 2018 [37]	France (Mulhouse)	Algorithm validation study	8 surgeons (novice, intermediate, expert)	da Vinci Surgical System; JIGSAWS dataset (suturing, needle passing, knot tying)	Kinematic data (76 vars @ 30Hz)	Expert skill level labels (novice, intermediate, expert)	1D CNN with domain-specific channel clustering; CAM	LOSO cross-validation; comparison to S-HMM, ApEn, SAX-VSM	Classification accuracy (micro and macro)	Comparison to benchmark methods on JIGSAWS	LOSO CV: 40 runs averaged	Heatmap visualisation of influential task segments via CAM	TensorFlow/Keras; Adam optimizer; Glorot initialisation	100% accuracy on suturing and needle passing; 93.2% on knot tying; interpretable feedback via CAM
Funke et al., 2019 [38]	Germany	Algorithm validation study	8 surgeons (novice, intermediate, expert)	da Vinci Surgical System; JIGSAWS dataset (suturing, needle passing, knot tying)	Video (RGB @ 10Hz; optical flow)	Expert-labelled skill level (novice, intermediate, expert)	I3D+TSN	LOSO CV; compared to kinematic-based methods	Accuracy, avg. recall, avg. F1 score	Benchmarking on JIGSAWS; comparison to state-of-the-art	4 runs averaged; pretrained on Kinetics	Implicit via classification	PyTorch; pretrained I3D on Kinetics; Adam optimizer	95.1-100% accuracy; video-only approach competitive with kinematic methods
Ebina et al., 2025 [39]	Japan/Hokkaido University Hospital	External validation study	52 participants (38 urologists, 4 residents, 10 students)	Box trainer (Endowork ProII) with porcine organs; dissection and suturing tasks	Optical MoCap (OptiTrack V120:Trio) at 120 Hz	Expert-evaluated GOALS scores; self-reported surgical caseload	PCA-SVM (classification), PCA-SVR (GOALS regression); SHAP for explainability	Expert ratings; self-reported experience tiers	Kinematic parameters (velocity, acceleration, jerk, path length, bimanual dexterity)	Correlation: GOALS 0.86-0.91; MAE: 1.56-2.51; classification accuracy: 56.9-67.3%	ICC for expert GOALS: 0.81-0.83; previously validated ML models	Real-time automated feedback with SHAP explanations, GOALS radar chart, and tier classification	MATLAB? Savitzky-Golay filtering; 120 Hz sampling	High user satisfaction (88% understandable, 75% acceptable); GOALS feedback is most valued
Brown and Kuchenbecker, 2023 [40]	USA/University of Pennsylvania	RCT	29 novices (24 med students, 5 residents)	da Vinci Standard robot; peg transfer task	Accelerometers, force sensor, video	Expert-rated GEARS scores (ground truth for algorithm training)	Regression-based ML algorithm (features from force, acceleration, time)	Control group (no feedback) vs. feedback group (automated GEARS scores)	Force integral, trial duration, tool acceleration, GEARS scores	No significant difference in performance metrics or learning rate between groups	Algorithm previously validated (Brown et al., 2017); high inter-rater reliability for GEARS	Post-trial automated GEARS scores (5 domains) without explanation	Custom data acquisition system; regression algorithm in R?	Feedback improved self-awareness but not skill acquisition; high user acceptance
Bogar et al., 2024 [41]	Hungary/University of Pécs	Single-blinded RCT	60 medical students (3rd year, no prior experience)	Custom VR simulator (Meta Quest 2) vs. FLS box trainer; peg transfer task	Video recording (box trainer); VR intrinsic data	Expert evaluation (6 professionals, FLS criteria)	YOLOv8 object detection+custom algorithm for pitfall/error detection	Control (box trainer) vs. VR group; AI vs. human raters	Exercise time, error detection (pitfalls), pass/fail rate	95% agreement AI vs. expert; no significant difference in training outcomes between groups	Cohen's kappa: 0.90 (AI vs. expert); 0.82 (inter-expert)	Automated scoring (time, errors); no real-time feedback	NVIDIA RTX 2080 Ti; PyTorch; OpenCV; 240 videos analysed	VR training is as effective as a box trainer; AI evaluation is faster (59.47s avg. saving per evaluation)
Belmar et al., 2023 [42]	Chile/Catholic University of Chile	Algorithm validation study	369 trainees (via digital platform)	Box trainer; BD and PT tasks	Video recordings	Expert evaluators (pass/fail based on time)	YOLOv4+U-Net (object detection, clamp tracking)	Expert evaluator consensus	Exercise time, object drops, pass/fail rate	Agreement: 93.02% (PT) and 79.69% (BD); kappa: 0.86 (PT) and 0.59 (BD)	High precision (98%) for clamp tracking; moderate to almost perfect agreement	Automated pass/fail output; no real-time feedback	Python, PyTorch; 400 BD+480 PT training videos	AI feasible for mass evaluation; high agreement for PT and moderate for BD
Atroshchenko et al., 2025 [43]	Denmark/Aalborg University Hospital	Feasibility study	19 surgeons (4 experts, 15 novices)	da Vinci Xi; porcine wet lab tasks (atrial closure, mitral stitches, ITA dissection)	Video (1920x1080, 30 FPS)	Temporal annotation (BORIS) for AR; GEARS and TBS scores for SA	CNN+LSTM for AR and SA	5-fold cross-validation; held-out test set	AR: accuracy 93%, F1 93%; SA: accuracy 56%, F1 44%	GEARS/TBS used for SA labelling	Single annotator; data split to prevent leakage	Post-procedural	Python 3.9; 256x256 frames; dropout, L2 reg	AR is feasible with limited data; SA needs more expert data
Anh et al., 2020 [44]	Australia/Monash University	Comparative analysis	8 surgeons (novice to expert)	da Vinci; JIGSAWS tasks (suturing, knot tying, needle passing)	Kinematic (76 motion signals @30Hz)	Expert-labelled skill level (novice, intermediate, expert)	Comparison of 9 feature extraction techniques+SVM	LOSO CV	Accuracy: CNN best (suturing: 96.84%; knot tying: 92.75%; needle passing: 95.36%)	JIGSAWS benchmark dataset	Code publicly available	Near real-time (4- 6s windows)	NVIDIA GTX1080Ti; shallow CNN/LSTM	CNN and PCA performed best; window size (4-6s) optimal
Alonso-Silverio et al., 2018 [45]	Mexico/university and hospital	Feasibility study	20 participants (4 experts, 6 residents, 10 students)	Custom box trainer; MISTELS tasks (transferring, pattern cutting)	Video (webcam)	Expert/non-expert classification	ANN (Levenberg-Marquardt)	Holdout, 10-fold CV, leave-one-out	Accuracy: 93.94%; AUC: 0.97; sensitivity: 0.78; specificity: 1.00	Construct (expert vs. novice)	Multiple validation schemes	Post-task	Raspberry Pi 2; Python/OpenCV	Low-cost
Akada et al., 2025 [46]	Japan/university and hospital	Pilot study	6 participants (3 experts, 3 novices)	Model eye; intrascleral IOL fixation (CP1, CP2)	Wearable strain sensors (C-Stretch)	Expert/novice classification	LightGBM on tsfresh features	3-fold cross-validation	CP1 accuracy: 87.9%; CP2 accuracy: 86.9%	Construct (expert vs. novice)	Cross-validation results	Not specified	Python (tsfresh, LightGBM); high-precision sensors	Thumb/index finger movements are key for skill differentiation; experts are faster

## References

[1] Feifer A, Al-Ammari A, Kovac E, Delisle J, Carrier S, Anidjar M (2011). Randomized controlled trial of virtual reality and hybrid simulation for robotic surgical training. BJU Int.

[2] Liss MA, McDougall EM (2013). Robotic surgical simulation. Cancer J.

[3] Chen IA, Ghazi A, Sridhar A, Stoyanov D, Slack M, Kelly JD, Collins JW (2021). Evolving robotic surgery training and improving patient safety, with the integration of novel technologies. World J Urol.

[4] Riddle EW, Kewalramani D, Narayan M, Jones DB (2024). Surgical simulation: virtual reality to artificial intelligence. Curr Probl Surg.

[5] Amparore D, Sica M, Verri P (2024). Computer vision and machine-learning techniques for automatic 3D virtual images overlapping during augmented reality guided robotic partial nephrectomy. Technol Cancer Res Treat.

[6] Brian R, Murillo A, Gomes C, Alseidi A (2024). Artificial intelligence and robotic surgical education. Global Surg Educ.

[7] Hatcher AJ, Beneville BT, Awad MM (2025). The evolution of surgical skills simulation education: robotic skills. Surgery.

[8] Andersen NL, Jensen RO, Konge L (2023). Immersive virtual reality in basic point-of-care ultrasound training: a randomized controlled trial. Ultrasound Med Biol.

[9] Haney CM, Kowalewski KF, Schmidt MW (2023). Robotic-assisted versus laparoscopic bowel anastomoses: randomized crossover in vivo experimental study. Surg Endosc.

[10] Andras I, Mazzone E, van Leeuwen FW (2020). Artificial intelligence and robotics: a combination that is changing the operating room. World J Urol.

[11] Nathan A, Patel S, Georgi M (2023). Virtual classroom proficiency-based progression for robotic surgery training (VROBOT): a randomised, prospective, cross-over, effectiveness study. J Robot Surg.

[12] Gani A, Pickering O, Ellis C, Sabri O, Pucher P (2022). Impact of haptic feedback on surgical training outcomes: a randomised controlled trial of haptic versus non-haptic immersive virtual reality training. Ann Med Surg (Lond).

[13] Fukuta A, Yamashita S, Maniwa J (2025). Artificial intelligence facilitates the potential of simulator training: an innovative laparoscopic surgical skill validation system using artificial intelligence technology. Int J Comput Assist Radiol Surg.

[14] Chen X, Liao P, Liu S, Zhu J, Abdullah AS, Xiao Y (2024). Effect of virtual reality training to enhance laparoscopic assistance skills. BMC Med Educ.

[15] Lakshmi AA, Vijayaraj A, Shree CK, Kumar S, Santhosh V, Tharun KS (2025). Artificial intelligence enhanced virtual reality surgery training. 2025 2nd International Conference on Research Methodologies in Knowledge Management, Artificial Intelligence and Telecommunication Engineering (RMKMATE).

[16] Igaki T, Kitaguchi D, Matsuzaki H (2023). Automatic surgical skill assessment system based on concordance of standardized surgical field development using artificial intelligence. JAMA Surg.

[17] Page MJ, McKenzie JE, Bossuyt PM (2021). The PRISMA 2020 statement: an updated guideline for reporting systematic reviews. J Clin Epidemiol.

[18] Sugiyama T, Tang M, Sugimori H, Sakamoto M, Fujimura M (2025). Artificial intelligence-integrated video analysis of vessel area changes and instrument motion for microsurgical skill assessment. Sci Rep.

[19] Singh R, Godiyal AK, Chavakula P, Suri A (2023). Craniotomy simulator with force myography and machine learning-based skills assessment. Bioengineering (Basel).

[20] Prevezanou K, Seimenis I, Karaiskos P, Pikoulis E, Lykoudis P M, Loukas C (2024). Machine learning approaches for evaluating the progress of surgical training on a virtual reality simulator. Appl Sci.

[21] Pisla D, Hajjar N A, Rus G (2025). Development of an augmented reality surgical trainer for minimally invasive pancreatic surgery. Appl Sci.

[22] Pan M, Wang S, Li J, Li J, Yang X, Liang K (2023). An automated skill assessment framework based on visual motion signals and a deep neural network in robot-assisted minimally invasive surgery. Sensors (Basel).

[23] Nakajima K, Takenaka S, Kitaguchi D (2025). Artificial intelligence assessment of tissue-dissection efficiency in laparoscopic colorectal surgery. Langenbecks Arch Surg.

[24] Nakajima K, Kitaguchi D, Takenaka S (2024). Automated surgical skill assessment in colorectal surgery using a deep learning-based surgical phase recognition model. Surg Endosc.

[25] Moglia A, Morelli L, D'Ischia R (2022). Ensemble deep learning for the prediction of proficiency at a virtual simulator for robot-assisted surgery. Surg Endosc.

[26] Mirchi N, Bissonnette V, Yilmaz R, Ledwos N, Winkler-Schwartz A, Del Maestro RF (2020). The virtual operative assistant: an explainable artificial intelligence tool for simulation-based training in surgery and medicine. PLoS One.

[27] Luongo F, Hakim R, Nguyen JH, Anandkumar A, Hung AJ (2021). Deep learning-based computer vision to recognize and classify suturing gestures in robot-assisted surgery. Surgery.

[28] Li L, Chen Z, Zaw TH, Luo B, Yang K, Wang X (2024). Skill assessment based on clutch use in cross-platform robot-assisted surgery. Surg Endosc.

[29] Lee D, Yu HW, Kwon H, Kong HJ, Lee KE, Kim HC (2020). Evaluation of surgical skills during robotic surgery by deep learning-based multiple surgical instrument tracking in training and actual operations. J Clin Med.

[30] Khanfar AF, Motamedi S, Safford SD, Moore J, Menold J, Miller S (2025). Visual attention and cognitive workload using different laparoscopic box trainers and mixed-reality feedback. Surg Endosc.

[31] Kasa K, Burns D, Goldenberg MG, Selim O, Whyne C, Hardisty M (2022). Multi-modal deep learning for assessing surgeon technical skill. Sensors (Basel).

[32] Karlik B, Yilmaz R, Winkler-Schwartz A, Mirchi N, Bissonnette V, Ledwos N, Del Maestro R (2021). Assessment of surgical expertise in virtual reality simulation by hybrid deep neural network algorithms. International Journal of Artificial Intelligence and Expert Systems (IJAE).

[33] Jokinen E, Mikkola TS, Härkki P (2020). Simulator training and residents' first laparoscopic hysterectomy: a randomized controlled trial. Surg Endosc.

[34] Ismail Fawaz H, Forestier G, Weber J, Idoumghar L, Muller PA (2019). Accurate and interpretable evaluation of surgical skills from kinematic data using fully convolutional neural networks. Int J Comput Assist Radiol Surg.

[35] Fazlollahi AM, Yilmaz R, Winkler-Schwartz A (2023). AI in surgical curriculum design and unintended outcomes for technical competencies in simulation training. JAMA Netw Open.

[36] Fazlollahi AM, Bakhaidar M, Alsayegh A (2022). Effect of artificial intelligence tutoring vs expert instruction on learning simulated surgical skills among medical students: a randomized clinical trial. JAMA Netw Open.

[37] Ismail Fawaz H, Forestier G, Weber J, Idoumghar L, Muller PA (2018). Evaluating surgical skills from kinematic data using convolutional neural networks. Medical Image Computing and Computer Assisted Intervention.

[38] Funke I, Mees ST, Weitz J, Speidel S (2019). Video-based surgical skill assessment using 3D convolutional neural networks. Int J Comput Assist Radiol Surg.

[39] Ebina K, Abe T, Hotta K (2025). External validation of a motion capture-based surgical skill assessment system in laparoscopic simulation training environments. Surg Endosc.

[40] Brown JD, Kuchenbecker KJ (2023). Effects of automated skill assessment on robotic surgery training. Int J Med Robot.

[41] Bogar PZ, Virag M, Bene M (2024). Validation of a novel, low-fidelity virtual reality simulator and an artificial intelligence assessment approach for peg transfer laparoscopic training. Sci Rep.

[42] Belmar F, Gaete MI, Escalona G (2023). Artificial intelligence in laparoscopic simulation: a promising future for large-scale automated evaluations. Surg Endosc.

[43] Atroshchenko GV, Korup LR, Hashemi N, Østergaard LR, Tolsgaard MG, Rasmussen S (2025). Artificial intelligence-based action recognition and skill assessment in robotic cardiac surgery simulation: a feasibility study. J Robot Surg.

[44] Anh NX, Nataraja RM, Chauhan S (2020). Towards near real-time assessment of surgical skills: a comparison of feature extraction techniques. Comput Methods Programs Biomed.

[45] Alonso-Silverio GA, Pérez-Escamirosa F, Bruno-Sanchez R, Ortiz-Simon JL, Muñoz-Guerrero R, Minor-Martinez A, Alarcón-Paredes A (2018). Development of a laparoscopic box trainer based on open source hardware and artificial intelligence for objective assessment of surgical psychomotor skills. Surg Innov.

[46] Akada M, Tabuchi H, Okamoto Y (2025). Quantitative assessment of microsurgical skill in intrascleral fixation surgery using wearable strain sensors: a pilot study. Transl Vis Sci Technol.

[47] Higgins JP, Altman DG, Gøtzsche PC (2011). The Cochrane Collaboration's tool for assessing risk of bias in randomised trials. BMJ.

[48] Sterne JA, Hernán MA, Reeves BC (2016). ROBINS-I: a tool for assessing risk of bias in non-randomised studies of interventions. BMJ.

[49] Yang JH, Goodman ED, Dawes AJ (2023). Using AI and computer vision to analyze technical proficiency in robotic surgery. Surg Endosc.

[50] Faber J, Fonseca LM (2014). How sample size influences research outcomes. Dental Press J Orthod.

[51] Topping KJ, Gehringer E, Khosravi H, Gudipati S, Jadhav K, Susarla S (2025). Enhancing peer assessment with artificial intelligence. Int J Educ Technol High Educ.

[52] Hooda M, Rana C, Dahiya O, Rizwan A, Hossain MS (2022). Artificial intelligence for assessment and feedback to enhance student success in higher education. Mathematical Problems in Engineering.

[53] Holzmeister F, Johannesson M, Böhm R, Dreber A, Huber J, Kirchler M (2024). Heterogeneity in effect size estimates. Proc Natl Acad Sci U S A.

[54] Kogan JR, Holmboe ES, Hauer KE (2009). Tools for direct observation and assessment of clinical skills of medical trainees: a systematic review. JAMA.

[55] Chan T, Sebok-Syer S, Thoma B, Wise A, Sherbino J, Pusic M (2018). Learning analytics in medical education assessment: the past, the present, and the future. AEM Educ Train.

